# Production and characterization of novel ssRNA bacteriophage virus-like particles from metagenomic sequencing data

**DOI:** 10.1186/s12951-019-0497-8

**Published:** 2019-05-13

**Authors:** Ilva Liekniņa, Gints Kalniņš, Ināra Akopjana, Jānis Bogans, Mihails Šišovs, Juris Jansons, Jānis Rūmnieks, Kaspars Tārs

**Affiliations:** Latvian Biomedical Research and Study Center, Rātsupītes 1, Riga, LV1067 Latvia

## Abstract

**Background:**

Protein shells assembled from viral coat proteins are an attractive platform for development of new vaccines and other tools such as targeted bioimaging and drug delivery agents. Virus-like particles (VLPs) derived from the single-stranded RNA (ssRNA) bacteriophage coat proteins (CPs) have been important and successful contenders in the area due to their simplicity and robustness. However, only a few different VLP types are available that put certain limitations on continued developments and expanded adaptation of ssRNA phage VLP technology. Metagenomic studies have been a rich source for discovering novel viral sequences, and in recent years have unraveled numerous ssRNA phage genomes significantly different from those known before. Here, we describe the use of ssRNA CP sequences found in metagenomic data to experimentally produce and characterize novel VLPs.

**Results:**

Approximately 150 ssRNA phage CP sequences were sourced from metagenomic sequence data and grouped into 14 different clusters based on CP sequence similarity analysis. 110 CP-encoding sequences were obtained by gene synthesis and expressed in bacteria which in 80 cases resulted in VLP assembly. Production and purification of the VLPs was straightforward and compatible with established protocols, with the only exception that a considerable proportion of the CPs had to be produced at a lower temperature to ensure VLP assembly. The VLP morphology was similar to that of the previously studied phages, although a few deviations such as elongated or smaller particles were noted in certain cases. In addition, stabilizing inter-subunit disulfide bonds were detected in six VLPs and several possible candidate RNA structures in the phage genomes were identified that might bind to the coat protein and ensure specific RNA packaging.

**Conclusions:**

Compared to the few types of ssRNA phage VLPs that were used before, several dozens of new particles representing ten distinct similarity groups are now available with a notable potential for biotechnological applications. It is believed that the novel VLPs described in this paper will provide the groundwork for future development of new vaccines and other applications based on ssRNA bacteriophage VLPs.

**Electronic supplementary material:**

The online version of this article (10.1186/s12951-019-0497-8) contains supplementary material, which is available to authorized users.

## Background

The single-stranded RNA (ssRNA) bacteriophages of the Levivirdae family are small viruses that infect a variety of Gram-negative bacteria. Their virions consist of a compact, approximately 3500 to 4200 nucleotide-long genome packaged in a small, spherical-looking protein shell about 28 nm in diameter with an underlying T = 3 quasi-equivalent icosahedral symmetry. The capsid is constituted of the major coat protein (CP) and one or two species of minor structural proteins that are involved in recognition and packaging of the genome and are required to adsorb the virion to the bacterial receptor and convey the RNA genome into the cell. At least for the currently studied ssRNA phages, the minor virion proteins are not essential either for assembly or for structural integrity of the protein shell, and recombinant expression of a cloned coat protein gene results in the appearance of virus-like particles (VLPs) that are morphologically very similar to native virions but have spontaneously packaged bacterial RNA inside the particles instead of the genome [1–4].

The ssRNA phage VLPs have found a variety of applications, mostly in the field of vaccine development where various antigens are presented onto the capsid surface to invoke a strong immune response. Phage Qβ VLPs conjugated with various peptide and small-molecule moieties have reached clinical trials against conditions such as hypertension [5], asthma [6] or smoking addiction [7], phage MS2 VLPs have been successfully used as carriers for epitopes from the human papilloma virus [8], while modified phage AP205 VLPs have shown promising results as vaccine candidates against West Nile virus [9]. The ssRNA phage VLP technology has been further extended for encapsulation of both macromolecular and small-molecule substances of interest inside the particles, which in combination with VLP surface modification allows for the development of targeted bioimaging and drug delivery agents (see [10, 11] for comprehensive reviews). ssRNA phage CPs and VLPs have also found a number of applications as tools for molecular biology research, notably in generation of peptide display libraries [12], identification of protein–RNA interactions [13, 14] and real-time imaging of RNA molecules in living cells [15].

Up to recently, the number of known ssRNA phages has been rather small. All of the currently identified phages use various Proteobacteria as their hosts; the great majority of these infect *Escherichia coli* and related Enterobacteria, while the remaining few target bacteria of the *Pseudomonas*, *Acinetobacter* or *Caulobacter* genera. The CPs of the Enterobacteria- and *Pseudomonas*-specific phages share very low, yet still detectable sequence similarity, while those from the *Acinetobacter* and *Caulobacter* phages, of which only a single representative of each has been sequenced, have no sequence similarity to other CPs [16, 17]. The different CPs vary considerably both in their overall amenability for modification and for tolerance of particular foreign sequences, as well as in their capability to recognize specific RNA for encapsulation and the stability of the assembled VLPs. Oftentimes, for a particular antigen a number of different VLP carriers and modification strategies have to be screened until a suitable one, if any, is found. In vaccine development and related areas, the immune response against the carrier coat protein has also to be taken into account, and a narrow range of available CPs adversely limits the number of potential vaccines that could be produced using the ssRNA phage platform. Discovery and characterization of novel ssRNA phage CPs and VLPs is therefore of considerable interest for continued developments in the area.

While no novel ssRNA phages have been isolated lately, the increasing metagenomic sequencing efforts in recent years have uncovered a previously unknown diversity of these viruses in nature. In 2015, genomes of two novel Leviviridae phages EC and MB were assembled from San Francisco wastewater [18], and soon a much wider study revealed over 150 partial ssRNA phage sequences in different RNA metagenomes [19]. A survey of RNA virus sequences from invertebrates resulted in more than 60 additional ssRNA phage genome sequences [20]. In the majority of the partial genomes, an open reading frame (ORF) between the conserved maturation and replicase genes can be identified that putatively encodes a coat protein, although the ORFs show great variation in length and sequence and in numerous cases no similarity to the known ssRNA phage CPs or any other proteins.

While the metagenomic studies have greatly expanded the known ssRNA phage diversity, infectious phages cannot be resurrected from the partial genome sequences, and their host bacteria, along with many other aspects of their biology, remain unknown. However, the CPs of the previously studied ssRNA phages have been able to assemble into VLPs in absence of other phage components, which provides an opportunity to obtain and study ssRNA phage VLPs even if the CP sequence is the only available information. In this study, we acquired 110 putative ssRNA phage CP-encoding ORFs from the metagenomic data using gene synthesis, and here we report the expression, purification and characterization of 80 novel ssRNA phage VLPs.

## Results

### CP similarity analysis

**Fig. 1:**
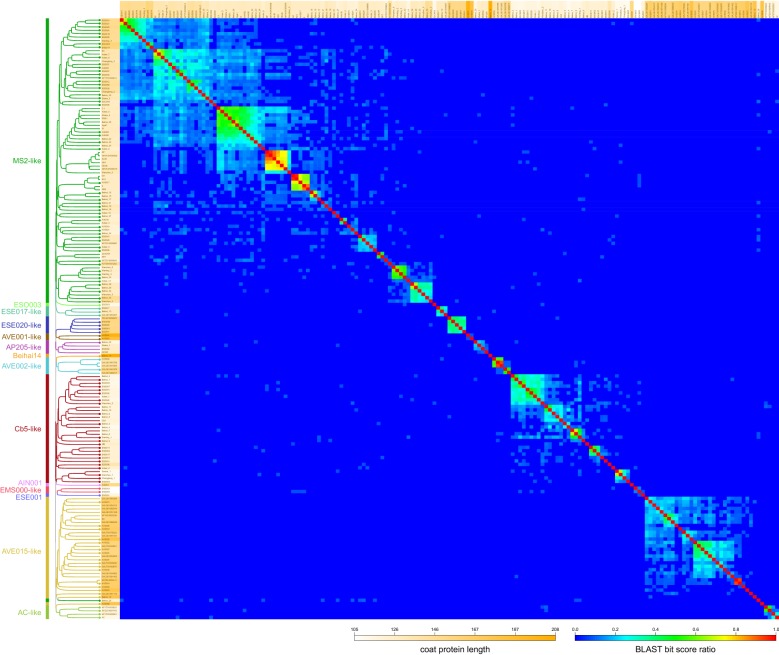
ssRNA phage CP similarity groups. All available CP sequences were compared in BLAST analysis followed by UPGMA clustering based on BLAST bit score ratios (hit score/self score). The resulting dendrogram and heat map representation of CP diversity are presented. The orange shading of CP labels corresponds to their length distribution. The CPs that were studied experimentally in this paper are indicated with dots at branch tips

Based on multiple sequence alignment, the previously known ssRNA phage CPs can be divided into three broad similarity groups represented by the Enterobacteria- and *Pseudomonas*-infecting phages, the *Acinetobacter* phage AP205 [16] and the *Caulobacter* phage Cb5 [17], respectively. To reassess the ssRNA phage CP diversity in light of the new data, we compiled for comparison a set of all of the published CP sequences together with some additional ones that could be located in NCBI’s nucleotide databases. However, for the CP sequences from the metagenomic data, a multiple sequence alignment was deemed unreliable due to the often very weak sequence similarity and broadly variant protein length that ranged from 105 to 208 residues compared to only 122 to 132 in the previously known phages. All available ssRNA phage CP sequences were therefore subjected to a BLAST similarity analysis, followed by UPGMA clustering based on BLAST bit score ratios (hit score/self score). The resulting clustering analysis and the resulting tree representation (Fig. 1), while not a proper phylogenetic reconstruction, provides useful information regarding the diversity of the novel CPs and their relatedness to the previously known phages.

Almost a half, or over 80, of the known ssRNA phage CP sequences cluster into an MS2-like supergroup with approximately ten recognizable subgroups. Two of those represent the currently recognized *Levivirus* and *Allolevivirus* F-pili specific phage genera, with an adjacent cluster containing the other conjugative-pili specific phages M [21], C-1 [22], PRR1 [23] and Hgal1 [22]. Two additional neighboring CP clusters are comprised solely of metagenomic sequences, with increasing CP length and decreasing CP similarity to the known phages. The supergroup includes some additional smaller clusters with low similarity to MS2 [24] or Qβ [4], one of which contains the previously known *Pseudomonas* phage PP7 [25]. A Cb5-like supergroup emerges as the second largest that includes the *Caulobacter* phage Cb5 and more than 30 related CP sequences from the metagenomic data. These further cluster into five subgroups, all formed by short and rather uniform-length proteins (most around 115–120 residues). One more supergroup with some 30 sequences emerges which is comprised entirely of metagenomic sequences and contains two major recognizable subgroups. The CPs in this “AVE015-like” supergroup, named after a representative phage from the group, are considerably bigger than those of MS2- or Cb5-like phages with an average length around 165 residues, with some of them spanning 180 residues. The three supergroups comprise approximately 80% of all of the known ssRNA phage CP sequences; the rest cluster into several considerably smaller clades ranging from two to five CPs, designated here the ESE017-like, ESE020-like, AP205-like, AVE001-like, AVE002-like, EMS000-like and AC-like groups. Several CP sequences (AIN001, ESE001, ESO003 and Beihai14) have no reliable assignment to any of the clusters and were considered orphans. (For brevity, phage names such as “Beihai levi-like virus 14” from [20] are referred to as “Beihai14” in this paper.)

### Expression of the novel CPs

Our BLAST analysis allowed to recognize approximately 14 distinct ssRNA phage coat protein types, which is a noticeable increase from the three CP types known before. We selected 110 CP sequences from the metagenomic data to cover all CP groups and represent maximum diversity both in sequence and in length, and obtained the sequences using gene synthesis to study them experimentally. Interestingly, in a few of the genome sequences, there were two or three predicted ORFs of similar length between the maturation and replicase genes. The predicted coat protein ORF was always the one immediately following the maturation gene, however, the other ORFs were also included for experimental characterization. All protein sequences used in the study are available in Additional file 1: Table S1.

All of the CP ORFs were initially expressed in *Escherichia coli* using a T7 promoter-driven system in standard conditions. The vast majority of the CPs were produced in the expected high levels and only in very few cases no expression was detected (Additional file 2: Figure S1). A subsequent solubility analysis (Additional file 2: Figure S2) however revealed that only about 60% of the CPs are at least partially soluble while the rest were found in inclusion bodies. In an effort to mitigate the issue, we expressed the insoluble proteins at 15 °C that indeed rendered 85% of the previously insoluble CPs at least partially soluble and only six remained in inclusion bodies also at the lower temperature. It can be noted that the few non-CP-encoding ORFs included in the analysis were either not expressed at all or were insoluble. Expression and solubility data are summarized in Table 1.

**Table 1:** Properties of experimentally studied ssRNA phage coat proteins

CP	Similarity group	Length	Cysteines		TR	Production	Solubility		VLPs			
			Positions	S–S			37 °C	15 °C	EM	Morphology	d, nm	Tm, °C
*MS2*	*MS2*	*129*			*++*	*+++*	*+++*	*n.d.*	*+++*	*T = 3*	28	70
*Qβ*	*MS2*	*132*	*74, 80*	*56*	*++*	*+++*	*+++*	*n.d.*	*+++*	*T = 3*	30	90
*PRR1*	*MS2*	*131*			*++*	*+++*	*+++*	*n.d.*	*+++*	*T = 3*	n.d.	75
*PP7*	*MS2*	*127*	*67, 72*	*56*	*++*	*+++*	*+++*	*n.d.*	*+++*	*T = 3*	27	90
AIN000	MS2	131			+	++	–	++	++	T = 3	n.d.	45
AIN002	MS2	132				+++	–	++	++	T = 3	n.d.	55
AIN003	MS2	140				+++	–	++	++	T = 3	28	55
AIN010	MS2	125				±	+	n.d.	–	n/a	n/a	n/a
AVE017	MS2	129			+	+++	+++	n.d.	++	T = 3	26	65
AVE019	MS2	123			+	+++	+++	n.d.	+	T = 3	27	50
AVE021	MS2	123			+	+++	++	n.d.	++	T = 3	30	60
Beihai16	MS2	128			+	+++	+++	n.d.	–	n/a	n/a	n/a
Beihai17	MS2	129				+++	+++	n.d.	++	T = 3, T = 1	26	55
Beihai18	MS2	131				+++	+++	n.d.	+	T = 3	29	50
Beihai19	MS2	134			+	+++	+++	n.d.	+	T = 3	28	55
Beihai21	MS2	126			+	+++	++	n.d.	+	T = 3	n.d.	40
Beihai23	MS2	130				+++	+	n.d.	+	T = 3	28	35
Beihai26	MS2	123			+	+++	+++	n.d.	+	T = 3	n.d.	50
Beihai27	MS2	122				+++	+++	n.d.	–	n/a	n/a	n/a
Beihai28	MS2	132			+	+++	+++	n.d.	++	T = 3	30	65
Beihai30	MS2	149				+++	++	n.d.	++	T = 3	n.d.	65
Beihai32	MS2	130			+	+++	++	n.d.	++	T = 3	31	70
Beihai33	MS2	126			+	+++	+++	+++	++	T = 3	28	n.d.
Beihai34	MS2	121				+++	+++	n.d.	+	T = 3	25	50
EMS014	MS2	156			+	+++	+	n.d.	+	T = 3	n.d.	n.d.
EOC000	MS2	129			+	+++	–	++	++	T = 3	n.d.	n.d.
EOC005	MS2	159				+++	–	++	+	T = 3	n.d.	60
ESE005	MS2	149			+	+++	–	–	n.d.	n/a	n/a	n/a
ESE006	MS2	129			+	+++	–	+++	++	T = 3	n.d.	n.d.
ESE007	MS2	137	93, 115	M		+++	–	+++	++	T = 3	30	90
ESE009	MS2	130				+++	–	+	–	n/a	n/a	n/a
ESE010	MS2	132				+++	+	n.d.	–	n/a	n/a	n/a
ESE012	MS2	143			+	+++	+	n.d.	+	T = 3	n.d.	n.d.
ESE019	MS2	150				+++	–	++	++	T = 3	32	50
ESE021	MS2	150			+	+++	–	+	+	T = 3	n.d.	50
ESE024	MS2	149				+++	–	++	+	T = 3	29	50
ESE025	MS2	152				+++	–	++	+++	T = 3	36	40
ESE029	MS2	132				+++	+++	n.d.	+++	T = 3	22	70
ESE030	MS2	142				+++	–	++	+++	T = 3	n.d.	35
ESE037	MS2	140			+	+++	–	+++	++	T = 3	n.d.	70
ESE046	MS2	137				+++	–	+++	++	T = 3	31	36
ESE058	MS2	146			+	+++	–	++	+++	T = 3	n.d.	55
ESO010	MS2	149			+	+++	–	+	–	n/a	n/a	n/a
Hubei2	MS2	124				+++	+	n.d.	–	n/a	n/a	n/a
Hubei3	MS2	128				+++	–	++	+	T = 3	n.d.	60
Hubei6	MS2	125			+	+++	+	n.d.	++	T = 3	24	60
Hubei8	MS2	137				+++	–	++	+	T = 3	26	40
Hubei10	MS2	129	76, 77	56		+++	+++	n.d.	++	T = 3	30	70
Hubei14	MS2	117			+	+++	+++	n.d.	+++	T = 3	28	70
NFYT01000214	MS2	135	78,79,95			+++	++	n.d.	++	T = 3	42	65
NFZC01009824	MS2	122	68, 70	n.d.		+++	+	n.d.	++	T = 3	n.d.	n.d.
Shahe3	MS2	136			+	+++	–	+	++	T = 3	29	50
Wenling2	MS2	128			+	+++	+++	n.d.	++	T = 3	28	70
Wenling3	MS2	126			+	+++	++	n.d.	+	T = 3	26	60
Wenzhou4	MS2	146			+	+++	–	++	+++	T = 3	30	50
AVE004	AVE015	159	107,108	n.d.	+	+++	+++	n.d.	+	T = 3	n.d.	n.d.
AVE006	AVE015	180				+++	++	n.d.	+	T = 3	n.d.	n.d.
AVE007	AVE015	156	46.134	N		+++	++	n.d.	++	T = 3, T = 1	30	n.d.
AVE022	AVE015	158	59, 136		+	+++	+	n.d.	–	n/a	n/a	n/a
AVE024	AVE015	156	39, 58	N	+	+++	++	n.d.	+	T = 3	n.d.	n.d.
AVE039	AVE015	158	56,89, 130	N	+	+++	++	n.d.	++	T = 3	n.d.	60
GALT01000492	AVE015	165	103,104, 105	56		+++	++	n.d.	+++	Heterogenous	48	70
GALT01093879	AVE015	163	101,102,103	56		+++	++	n.d.	++	T = 3	30	75
AVE000	AVE015	167	61,98,99	N		+++	–	+++	+++	T = 4?	38	n.d.
AVE005	AVE015	164	94, 112	N	+	+++	–	+++	+	T = 3	40	70
AVE015	AVE015	167			+	+++	–	++	++	T = 3	n.d.	65
AVE016	AVE015	166	113, 158	N		+++	+++	n.d.	++	T = 3, some elongated	30	95
AVE018	AVE015	169	9, 43, 92	D	+	+++	+++	n.d.	+	T = 3	28	70
AVE020	AVE015	180	38, 146			+++	++	n.d.	–	n/a	n/a	n/a
AVE023	AVE015	177			+	+++	–	–	n.d.	n/a	n/a	n/a
Beihai12	AVE015	178			+	+++	+++	n.d.	–	n/a	n/a	n/a
GALQ01044112	AVE015	164	8,59,113	N		+++	+++	n.d.	++	T = 3	32	80
AVE002	AVE002	140			+	+	+	n.d.	+++	T = 3	31	65
GALQ01040378	AVE002	151				–	–	–	n.d.	n/a	n/a	n/a
Beihai13	ESE017	132				+++	–	+	++	T = 3	n.d.	n.d.
ESE017	ESE017	132				+++	+	n.d.	+	T = 3	27	70
GALQ01034907	ESE017	131				++	+	n.d.	+	T = 3	27	60
*AP205*	*AP205*	*130*	*64, 68*	*56*		*+++*	*+++*	*n.d.*	*+++*	*T = 3, T = 1*	28	75
Beihai20	AP205	117				+++	++	n.d.	–	n/a	n/a	n/a
*Cb5*	*Cb5*	*122*				*+++*	*+++*	*n.d.*	*+++*	*T = 3*	28	70
EMS002	Cb5	123	64, 68		+	+++	–	++	–	n/a	n/a	n/a
EMS011	Cb5	123	64.68	56		+++	–	++	++	T = 3	33	55
EMS017	Cb5	123	64, 68			–	–	n.d.	n.d.	n/a	n/a	n/a
ESE016	Cb5	123				+++	++	n.d.	–	n/a	n/a	n/a
ESO001	Cb5	124			+	+++	–	++	++	T = 3	40	n.d.
Beihai3	Cb5	119				+++	±	n.d.	+	T = 3	24	60
Beihai6	Cb5	119			+	+++	+++	n.d.	–	n/a	n/a	n/a
MB	Cb5	127				++	–	++	–	n/a	n/a	n/a
Beihai1	Cb5	116			+	++	+	n.d.	+	T = 3	n.d.	n.d.
EMS007	Cb5	116			+	+++	±	n.d.	–	n/a	n/a	n/a
ESE008	Cb5	138			+	+++	–	+++	–	n/a	n/a	n/a
ESE015	Cb5	119				+++	+	n.d.	+	T = 3	31	55
ESE022	Cb5	116			+	+	–	++	–	n/a	n/a	n/a
ESE026	Cb5	119			+	+++	+++	n.d.	+	T = 3	n.d.	50
ESO000	Cb5	115			+	+++	–	++	–	n/a	n/a	n/a
Wenzhou2	Cb5	122				+++	±	n.d.	++	T = 3	26	60
Beihai9	Cb5	123			+	+++	+	n.d.	++	T = 3	29	50
Wenling1	Cb5	116				+++	+	n.d.	–	n/a	n/a	n/a
Changjiang1	Cb5	112			+	+++	+	n.d.	+	T = 3	n.d.	n.d.
EMS005	Cb5	112			+	+++	++	n.d.	–	n/a	n/a	n/a
Wenzhou1	Cb5	113			+	+++	+	n.d.	++	n/a	n/a	n/a
EMM000	ESE020	155			+	+++	–	++	+	T = 3	23	50
EMS001	ESE020	154				+++	+	n.d.	++	T = 3	n.d.	65
ESE020	ESE020	153			+	+++	+++	n.d.	+	T = 3	36	65
ESE041	ESE020	155			+	+++	–	+++	+	T = 3	34	50
AC	AC	115				+++	–	+++	++	T = 3	n.d.	50
NFYT01000391	AC	123	66,67,75	N		+++	+	n.d.	++	T = 1	18	75
NFZC01007443	AC	119	64, 66	5		+++	++	n.d.	++	T = 1	19	60
EMS000	EMS000	105	45, 48		+	+	++	n.d.	–	n/a	n/a	n/a
EMS003	EMS000	106				+++	–	+	–	n/a	n/a	n/a
AVE001	AVE001	202	82,83,84		+	+++	–	–	n.d.	n/a	n/a	n/a
AVE003	AVE001	183	88,90,176		+	+++	–	–	n.d.	n/a	n/a	n/a
Beihai14	Beihai14	208			+	+++	–	++	++	T = 3	31	85
ESE001	ESE001	118	61, 66	56		+++	±	n.d.	++	T = 3	30	75
ESO003	ESO003	113			+	+++	+	n.d.	++	T = 3	29	60
AIN001	AIN001	155			+	++	–	–	n.d.	n/a	n/a	n/a

Some previously studied CPs are included for reference and shown in italic. The listed properties include the assigned CP similarity group, length, presence of a translational repressor stem-loop (TR) in the genome (+, a putative hairpin structure predicted; ++, an experimentally confirmed TR), positions of cysteine residues in the protein if more than one is present, disulfide bonds in VLPs (56, covalently linked pentamers and hexamers; 5, pentamers; D, dimers; N, no disulfides detected), production level (+++, high; ++, average; +, low; ± , very low; –, not detected), solubility at 37 °C and 15 °C (+++, highly soluble; ++, at least 50% soluble; +, less than 50% soluble; –, completely insoluble), VLP formation by EM: (+++, highly efficient VLP formation; ++, reasonably good VLP formation; +, some detectable VLPs; –, no VLPs observed), characterization of VLP morphology, particle diameter from DLS measurements, and their “melting” temperature (thermal stability). n/a: not applicable due to lack of VLPs, n.d.: not determined

### Purification and characterization of VLP morphology

**Fig. 2:**
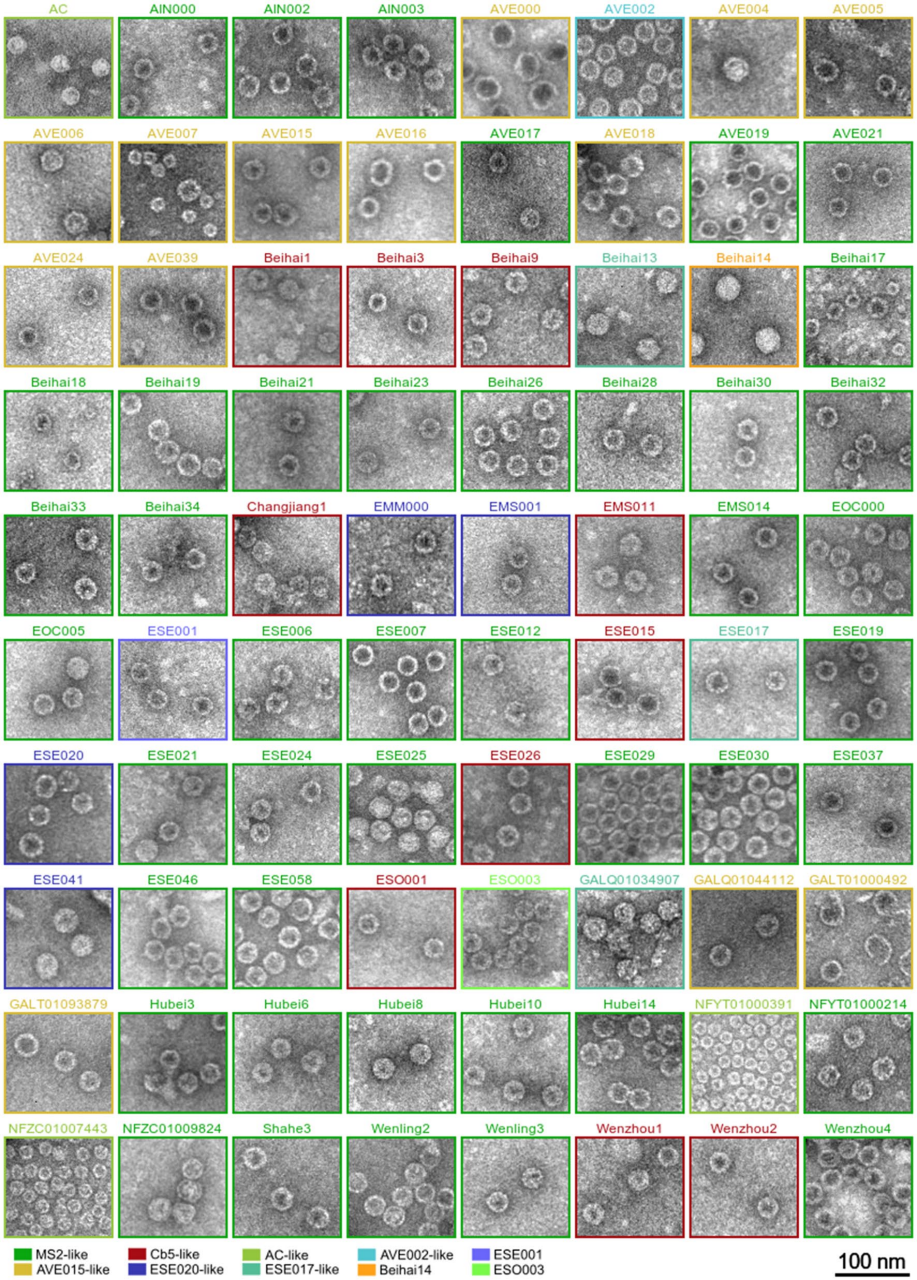
Representative electron micrographs of the produced VLPs. The constituent CP similarity groups as of Fig. 1 are indicated

After CP production, the crude *E. coli* lysates were separated by gel filtration and the fractions analyzed for CP presence in the expected molecular weight range for VLPs. In total 80, or approximately 72%, of the soluble CPs assembled into VLPs as confirmed by electron microscopy (Fig. 2). In the majority of cases, the VLP morphology resembled that of the previously characterized ssRNA phage VLPs with an apparent spherical shape 28 to 30 nm in diameter that corresponds to a T = 3 icosahedral particle. However, notable deviations from the standard particle size and shape were not uncommon. The VLPs formed by the AVE000 CP were noticeably bigger, reaching 35 to 40 nm in diameter which could correspond to a T = 4 icosahedral particle, but the preparation was rather heterogeneous with many elongated, squashed or incomplete particles. A somewhat similar view was observed also in GALT01000492 VLP preparations where the T = 3 particles were present in minority while the field of view was dominated by bigger VLP-resembling irregular objects. NFYT01000391 and NFZC01007443 CPs assembled into small particles approximately 18 nm in diameter with a presumed T = 1 icosahedral symmetry. In some other cases, two distinct VLP morphologies were present in the sample: a sizeable proportion of AVE016 VLPs appeared to have an elongated shape, while AVE007 and Beihai17 VLP preparations contained a mixture of T = 3 and T = 1 particles.

To further characterize the VLPs, we used dynamic light scattering (DLS) to determine the average particle size in solution. In a homogeneous sample, the particle size measured using DLS is in good agreement with values determined using other methods such as electron microscopy or X-ray crystallography, while significant deviations are an indicator of particle aggregation. For the majority of VLPs, the determined average particle diameter values (Table 1) lie within a range of 25–30 nm which is in good agreement with the size observed in EM. Using DLS, the NFYT01000391 and NFZC01007443 VLPs measured 18 to 19 nm in diameter which corresponds to the apparent T = 1 particles detected in EM, and likewise the bigger AVE000 VLPs measured 38 nm in diameter and the apparently heterogenous GALT01000492 preparation had an average particle diameter of 48 nm. VLPs with significant discrepancies between the EM and DLS data, such as NFYT01000214 with an observed diameter of 28–30 nm in EM but a measured size of 42 nm using DLS, or VLPs for which no reasonable estimate could not be obtained, likely indicate significant amount of aggregation in the samples.

### Stabilizing disulfide bonds in the novel VLPs

Coat protein modifications introduced for vaccine development and related applications have a tendency to destabilize the assembled VLPs, and the experimental success rate appears to positively correlate with the stability of the starting unmodified particles. While in some of the studied ssRNA phages inter-subunit contacts are mediated solely by non-covalent protein–protein interactions, in others coordinated metal ions [26, 27] and protein–RNA interactions [27] have been found that contribute to particle stability. In yet other phages such as Qβ [28], PP7 [29] and AP205 [30] the CP subunits are covalently linked together with disulfide bonds. The disulfides markedly increase the particle stability and have been a substantial factor in the advancement of Qβ and AP205 VLPs as the most successful ssRNA phage-derived carriers. Screening for stabilizing disulfide bonds in the novel VLPs is therefore of interest for selecting the best candidates for future VLP carriers.

**Fig. 3:**
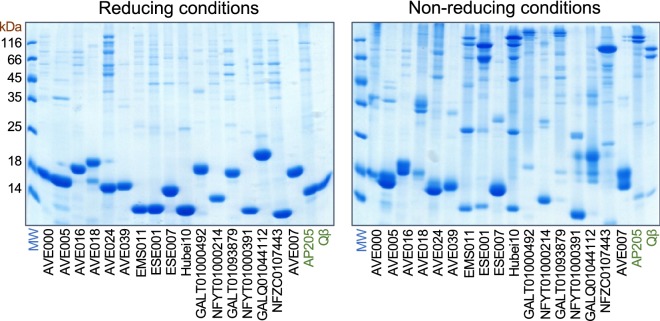
Disulfide bonds in the produced VLPs. The VLPs were subjected to denaturing and reducing or non-reducing conditions and analyzed in SDS polyacrylamide gel electrophoresis

In all of the previously studied ssRNA phage particles where disulfide bonds exist, they are formed between CP loops positioned around the icosahedral threefold and fivefold symmetry axes that results in covalently linked CP pentamers and hexamers in the capsid. It cannot be excluded, however, that in other phages stabilizing disulfide bonds might occur also in other positions. We therefore selected all experimentally available CPs that were able to assemble into VLPs, could be purified to near homogeneity and which contained at least two cysteine residues, and subjected the VLPs to denaturing but non-reducing conditions. In such conditions the disulfide-containing Qβ and AP205 VLPs disassemble into pentameric and hexameric CP species that can be tracked in SDS–polyacrylamide gel electrophoresis (Fig. 3). From the 17 tested novel VLPs, only EMS011, ESE001, Hubei10, GALT01000492 and GALT01093879 produced a pair of bands corresponding to the expected pentameric and hexameric species (Fig. 3); in most cases, a number of lower molecular weight complexes could also be discerned, suggesting that not all of the possible disulfide bridges have been formed in the VLPs. All of these five CPs contain cysteine residues located similarly to Qβ or AP205 approximately in the middle of the sequence; the EMS011 and ESE001 CPs contain two cysteine residues five or six positions apart, while the Hubei10 CP has two and GALT01000492 and GALT01093879 have three consecutive cysteine residues. In the latter two CPs, apparently only two of the residues are involved in inter-subunit contacts, although different pairs of cysteine side chains might be involved in making pentameric and hexameric contacts. From the tested proteins also the NFZC0107443 CP contains two similarly located cysteine residues three positions apart, but in non-reducing conditions the VLPs resolved into only a single higher molecular weight species. This is however consistent with the assumed T = 1 icosahedral structure of the NFZC0107443 VLPs from EM data as T = 1 particles involve only pentameric but no hexameric interactions. The rest of the VLPs did not produce apparent hexameric or pentameric species, however, AVE018 VLPs appeared to contain another kind of higher molecular weight covalent species putatively corresponding to a disulfide-linked CP dimer.

### VLP thermal stability

The thermal stability of a VLP is an important characteristic that positively correlates with the overall robustness of the particle and its performance in downstream applications such as vaccine development or drug delivery. To determine the thermal stability of the novel ssRNA phage VLPs, we subjected the particles to increasing temperatures and visualized their disassembly in native agarose gel electrophoresis. Intact ssRNA phage VLPs migrate as a distinct band in the gel which is detectable both when staining for RNA and for protein, but as the VLPs are heated and gradually disassemble, the VLP band accordingly becomes weaker until it completely disappears. The thermal stability Tm is defined as the lowest tested temperature at which the VLP band can no longer be observed.

The novel VLPs have a broad range of thermal stability (Table 1). While the majority (~ 77%) of the tested VLPs disassembled between 50 °C and 70 °C, a few were extremely unstable and were destroyed even at 35 °C, and some others had an unusually high Tm of up to 95 °C. VLPs of the previously studied ssRNA phages without inter-subunit covalent bonds typically disassemble at 60 to 70 °C [26, 27, 31] while those containing disulfides have a notably higher melting temperature of 75 to 95 °C. Interestingly, none of the newly characterized VLPs with the highest melting temperatures (AVE016, 95 °C, ESE007, 90 °C, Beihai14, 85 °C) have disulfide bonds between the subunits while the five VLPs with experimentally detected inter-subunit disulfides exhibit a relatively modest thermal stability between 55 and 75 °C. These results undermine the prior belief that inter-subunit disulfide bonds are a necessity for robust ssRNA phage VLPs and demonstrate that very stable particles can be built solely by non-covalent interactions. Further investigations are underway to determine the functional basis for the unusual stability of these VLPs.

### Potential CP–RNA interactions

In a number of the previously studied ssRNA phages, the coat protein recognizes and binds a genomic RNA hairpin at the beginning of the replicase gene which regulates the synthesis of the replicase enzyme and contributes to specific packaging of phage genome into the virions. The hairpin is a stem-loop structure comprised of an approximately eight base pair-long stem with an unpaired adenosine residue and a three- to six-nucleotide-long loop, and is often designated the translational operator (TR) of the replicase gene (see [32] for a review). The TR can be appended to an RNA molecule of choice as a tag where it can direct packaging of specific RNA molecules inside VLPs or serve for identifying protein–RNA interactions or tracking of RNA molecules in a living cell (see [10] for a review). Currently two distinct CP RNA binding modes are known for the ssRNA phages, the first shared by the conjugative pili-specific phages MS2 [33], PRR1 [34] and Qβ [35], and the other one found in the *Pseudomonas* phage PP7 [36]. No CP-TR binding has been detected in the more distantly related phages AP205 and Cb5 despite considerable effort, suggesting that the interaction is not universally conserved among the ssRNA phages.

**Fig. 4:**
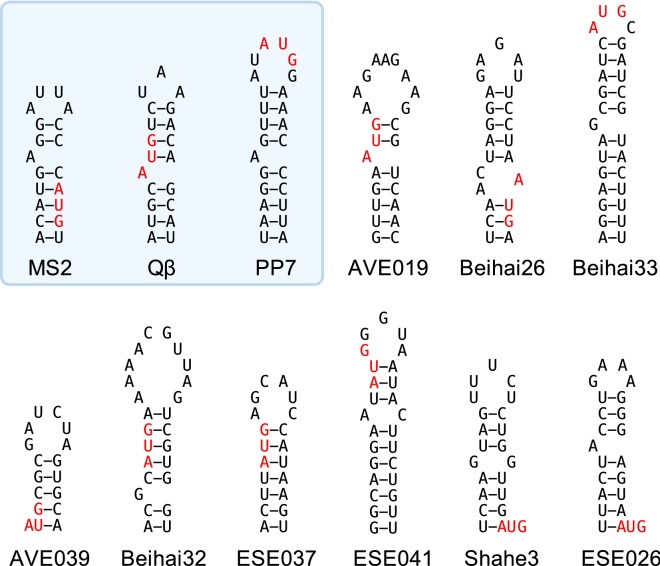
Putative TR hairpins found in the metagenomic ssRNA phage sequences for some of the CPs used in this study. The known TR hairpins of MS2, Qβ and PP7 phages are shown for reference. The initiation codon of the replicase gene is shown in red

The CP ability to specifically bind RNA is a certain advantage for VLP and other potential applications, therefore for our subset of experimentally available CPs we surveyed the corresponding genome sequences for possible TR hairpins at the beginning of the replicase ORFs. In the majority of cases, a putative stem-loop structure around the replicase initiation codon could indeed be detected. A number of examples are compiled in Fig. 4; all predictions are provided in Additional file 2: Figure S3 and are summarized in Table 1. Within the MS2-like CP supergroup there appears to be a trend that phages with CP sequences relatively more similar to those of MS2, PRR1 or Qβ also contain a TR-resembling hairpin in the genome, while for more distant phages the TR structures look increasingly dubious. A notable exception is a small cluster of Beihai33, Wenling2 and Wenling3 CPs which have very weak similarity to either the Ms2, Qβ or PP7 CPs, yet all of them have a prominent hairpin with a tetranucleotide sequence AUGC in the loop. In addition, despite differences in sequence, the base pairing in the stem has been preserved, suggesting that the hairpins are evolutionary conserved and might function as TRs through a possibly novel RNA binding mechanism to the two already known. No analogous structural conservation is observed among related phages in other CP similarity groups, which renders the function of the predicted hairpins as TRs somewhat questionable. However, affinities of the predicted TR hairpins for the respective CPs have to be experimentally determined, and discovery of additional protein–RNA interactions is clearly possible.

## Discussion

In the current study we have analyzed over 100 novel ssRNA phage CPs with the main objective to find candidates for development of future VLP carriers. A number of properties for the CPs and VLPs are desirable for these purposes: (1) high-level CP expression in bacteria; (2) efficient assembly of the CP into VLPs; (3) high stability of the assembled VLPs; (4) simple and effective means of VLP purification and (5) VLP tolerance for chemical and genetic modification. An ability to package substances of interest inside the VLPs is further preferable. In our study we have addressed a number of these points, and the sample size allows for some general conclusions to be made.

For all of the previously studied ssRNA phages, the CPs could be cloned and expressed in *E. coli* in standard conditions which resulted in highly efficient assembly of VLPs. We adopted a similar strategy for the novel ssRNA phage CPs, and the high-level expression part indeed did not present any problems with only a few of the proteins not being produced. Instead, the solubility of the produced CPs proved to be the first roadblock as in the initial conditions approximately 40% of the tested proteins were found in inclusion bodies. In this respect, it can be noted that the reported sources for the metagenomic datasets are extremely diverse, ranging from intestinal contents of warm-blooded animals to deep sea microbial sediments and arctic soils in Svalbard. It is therefore conceivable that at least for some of the proteins, high-level expression at 37 °C is such a dramatic departure from the conditions in their native host bacteria that it adversely affects their folding, stability or assembly into VLPs. This assumption appeared to be correct as lowering the CP expression temperature indeed largely resolved the solubility issues.

As the study progressed, it was further established that the solubility of the CP does not necessarily lead to VLP formation. Generally, CPs of the MS2-like supergroup readily formed VLPs although there were some exceptions in particular subclusters. CPs of the AVE015-like supergroup were also generally able to assemble into VLPs, although a higher prevalence of aberrant particles was observed in these samples. In contrast, in the Cb5-like supergroup VLP formation was detected for less than a half of the examined CPs. It can be noted that the Cb5 VLPs are very sensitive to salt [27, 37] which might translate also to related CPs, and it is possible that the presence of salt in buffer solutions or during the EM staining procedure might have triggered VLP disassembly. While the issue could perhaps be alleviated by taking extra care not to expose these VLPs to salt at any point, such experiments were not attempted as particles this unstable would not be of much interest for subsequent biotechnological developments. From the smaller CP similarity clusters, all expressed CPs from the AVE002-like, ESE017-like, ESE020-like and AC-like CP groups also formed VLPs. From the remaining CPs, only those from the, Beihai14, ESE001 and ESO003 phages were able to assemble into particles.

The rather high proportion of assembly-deficient CPs was somewhat unexpected from our prior experience and, besides the possible VLP stability issues discussed above, likely has several additional reasons. The metagenomic data vary significantly in quality and in some cases the failure of a CP to be expressed or to assemble into VLPs might result from an incorrect sequence caused by sequencing or sequence assembly errors. In other cases, the high-level heterologous expression in *Escherichia coli* might cause issues for CPs from phages with markedly different original hosts that might be more complex than growth temperature alone. In most of the cases, however, the failure to form VLPs is presumably caused by the absence of other phage components during the assembly process. The assumption that the presence of unspecific RNA is sufficient to promote particle assembly has been built on a small subset of previously studied coat proteins, and there is no particular reason to expect similar properties for all ssRNA phages. Contrary to the large DNA phages, the ssRNA phages do not package their genome into a preformed empty capsid but instead the CP subunits condense around the genomic RNA molecule to form an enclosing protein shell. In the process, the phage maturation protein specifically binds both the genome and the coat protein, and the genomic RNA itself has a highly complex three-dimensional shape that is thought to actively promote its encapsidation [38–41]. Considering that the biological function of the CP is to build virions and not VLPs, it is conceivable that some phages might have evolved to rely on the maturation protein and/or the full-length genome for assembly more than others. This would in turn manifest in the observed incapability of the CP to assemble into VLPs when expressed separately from the other phage components. Also, in other RNA viruses, specific RNA packaging signals have been described (see [42] and references therein), and it cannot be excluded that in some ssRNA phage genomes yet unidentified RNA structures exist that are crucial for assembly. Experimental verification of such possibilities is however difficult or impossible in absence of the actual phage that can be studied in the laboratory.

The purification of the novel VLPs was generally straightforward and in most cases, a previously established two-step VLP purification procedure using gel filtration and ion exchange chromatography was able to yield an at least 90% pure preparation. In some cases, however, only about 50% pure material was obtained, presumably due to low VLP stability and/or co-aggregation with bacterial proteins. Still, for the great majority of the novel VLPs, purification does not pose any problems and is suitable for biotechnological processes.

To further characterize the VLPs and enable rational structure-guided modification of the capsid, efforts are currently underway to determine their high-resolution three-dimensional structures by X-ray crystallography. The tolerance for foreign antigens by chemical and genetic modification is also being tested in our laboratory for a number of VLPs, and preliminary data indicate that several could be of comparable or superior performance to the Ms2, Qβ and AP205 VLPs (to be published).

## Conclusions

In this study, we have demonstrated that environmental viral sequences uncovered in metagenomic studies can be useful not only for comprehending the diversity of viruses in nature but can also be successfully utilized to reconstruct virus-like particles in a laboratory setting. In this way we have for the first time experimentally characterized 11 new ssRNA phage coat protein types and their ability to assemble into VLPs. The 80 novel ssRNA bacteriophage VLPs that we have obtained and characterized here will be important for development of new vaccines and related applications using the ssRNA phage VLP platform. The results also provide a rich ground for further fundamental studies of ssRNA bacteriophage biology such as their structure and protein–RNA interactions.

## Methods

### CP similarity analysis and clustering

The metagenomic ssRNA phage genome sequences were fetched from GenBank using accession numbers reported in [18–20] or sourced from supplementary data from [19]. An additional search for new ssRNA phage sequences was performed in January 2018 by querying the NCBI’s nucleotide (nt) and environmental nucleotide (env_nt) sequence databases with all available ssRNA phage protein sequences using the tblastn program from the BLAST + package [43]. The additional ssRNA phage protein sequences extracted from the hits were iteratively used in repeated queries until no new sequences were detected. A total of 31 additional CP sequences were recovered.

All available ssRNA phage CP sequences were used to generate a BLAST database against which each sequence was individually queried using the blastp program. The results in CSV format were imported into a Google Sheets document for calculation of BLAST bit score ratios (BSRs; the BLAST bit score of the hit divided by the bit score of the query sequence matched against itself) and creation of a distance matrix using values of 1-BSR as the distance measure. The matrix was used to cluster the sequences with the UPGMA algorithm using the program neighbor from the Phylip package [44]. Figtree v 1.4.3. [45] was used for visualization of the resulting tree. The data from the clustering analysis together with the BSR values were used for generating a heat map of CP variation in Google Sheets using Google Apps Script scripting facilities.

### CP expression

The CP-encoding sequences were synthesized by General Biosystems and provided by the manufacturer cloned in pET24a (Novagen) as expression-ready constructs.

For small-scale expression and solubility analysis, *E. coli* BL21(DE3) cells were transformed with CP-encoding plasmids, individual colonies were inoculated in 5 ml of LB media supplemented with 30 μg/ml kanamycin and incubated at 37 °C overnight without shaking. The overnight cultures were transferred into 50 ml of 2xTY medium and the cells were grown at 37 °C or 15 °C with aeration until OD_600_ reached 0.6 to 0.8. IPTG was then added to a final concentration of 1 mM and the cultures were incubated for additional 4 h at 37 °C or 20 h at 15 °C, after which aliquots were harvested by centrifugation for assessment of expression level and solubility by SDS-PAGE. Large-scale production for VLP purification purposes followed the same protocol using 2 l of 2xTY medium. To determine the solubility of the produced CPs, the aliquoted cells were suspended in lysis buffer (50 mM tris–HCl pH 8.0, 150 mM NaCl, 0.1% Triton X100, 1 mM PMSF) in a wet cell weight/buffer volume ratio of 1:4, lysed by sonication, centrifuged for 30 min at 13000 g and the supernatant and pellet analyzed in SDS-PAGE. The same protocol was used for preparation of lysates for VLP purification.

### VLP purification

For small-scale purification, 1 ml of clarified bacterial lysate was loaded onto a 12 ml, 6.6 × 400 mm Sepharose 4 FF column (GE Healthcare) equilibrated with PBS. Chromatography was done on an Acta Prime Plus system (GE Healthcare) with the flow rate set to 0.3 ml/min and fraction size to 1 ml. VLP-containing fractions were detected in SDS-PAGE and those of the highest purity were pooled and applied to a 0.7 ml, 6.6 × 50 mm Fractogel DEAE (M) ion exchange column (GE Healthcare). The flow-through was collected and the column further washed with 2 ml of PBS. Column-bound proteins were eluted with a linear 10 column volume gradient to PBS containing 1 M NaCl using a flow rate of 1 ml/min and fraction size of 1 ml on an Akta Pure 25 system (GE Healthcare). The VLPs were usually found in most of the fractions while the contaminating proteins only in some. The fractions of the highest purity were pooled, dialyzed against 20 mM tris–HCl pH 8.0, supplemented with glycerol to a final concentration of 50% and stored at − 20 °C for downstream experiments. The purification protocol was accordingly upscaled if a larger quantity of VLPs was required.

### Electron microscopy

For transmission electron microscopy, VLP samples after gel-filtration were adsorbed on carbon-Formvar-coated copper grids and negatively stained with a 1% aqueous solution of uranyl acetate. The grids were examined in a JEM-1230 electron microscope (JEOL Ltd., Tokyo, Japan) operated at 100 kV. Electron micrographs were recorded with iTEM software (version 3.2, Soft Imaging System GmbH) using a side-mounted Morada digital camera (Olympus-Soft Imaging System GmbH, Munster, Germany).

### Dynamic light scattering

DLS measurements were performed using Malvern Zetasizer Nano ZS and quartz cuvette ZEN2112, 173 Backscatter according to manufacturer’s instructions.

### Detection of disulfides in VLPs

Aliquots of purified cysteine-containing VLPs in storage buffer (20 mM Tris–HCl pH 8.0, 50% glycerol) were mixed with an equal volume of Laemmli buffer (0.125 M Tris–HCl pH 6.8, 20% glycerol) with or without added 5% 2-mercaptoethanol. The samples were heated for 10 min at 95 °C and run on a 15% polyacrylamide gel using a standard Tris–glycine SDS electrophoresis system.

### Determination of VLP thermal stability

Assessment of thermal stability was done essentially as described before [27]. VLP samples at a concentration of 1 mg/ml in 20 mM tris–HCl, pH 8.0 were heated for 15 min in a Veriti thermal cycler (Applied Biosystems) in a 5 °C-increment step gradient and then loaded on a 1% agarose gel. After electrophoresis in TAE buffer, the RNA was visualized with ethidium bromide and protein with Coomassie blue.

### Prediction of TR hairpins

The starting positions of replicase ORFs in the metagenomic ssRNA phage genome sequences were located based either on the available genome annotations or found by examining the sequences manually. A region flanking 20 nucleotides in both directions from the first nucleotide of the replicase ORF was extracted from each genome and the sequences were input to rnafold using the default parameters.

## **Additional files**

**Additional file 1: Table S1.** Protein sequences used in the study. Accession numbers are provided for the respective genome sequences. Entries without an accession number were sourced from Dataset S1 from Krishnamurthy et al. [19]. In cases where more than one open reading frame was found between the maturation and replicase genes, the coat protein corresponds to ORF2.

**Additional file 2: Figure S1.** SDS-PAGE analysis of the production level of the metagenomic ssRNA phage CPs. The samples represent total cellular protein content four hours after the CP expression was induced at 37 °C. 1 and M2—protein molecular weight markers (M1: bands of 10, 15, 25, 35, 40, 55, 70, 100, 130 and 180 kDa; M2: bands of 14, 18, 25, 35, 45, 66 and 116 kDa). **Figure S2.** Solubility of the metagenomic ssRNA phage CPs at 37°C and 15°C. The samples represent the soluble (s) and insoluble (d) cellular protein fractions after the CPs were produced at the indicated temperature. The proteins were produced at 15 °C only if they were completely insoluble at 37 °C. M2—lanes with MW marker—14, 18, 25, 35, 45, 66 and 116 kDa. M1 and M2—protein molecular weight markers (M1: bands of 10, 15, 25, 35, 40, 55, 70, 100, 130 and 180 kDa; M2: bands of 14, 18, 25, 35, 45, 66 and 116 kDa). **Figure S3.** Predicted RNA hairpin structures at the beginning of the replicase gene in the metagenomic ssRNA phage genome sequences. A region flanking 20 nucleotides in each direction from the first nucleotide of the replicase gene was used for the prediction.

## Data Availability

All data generated or analyzed during this study are included in this published article and its Additional files.

## References

[1] Kastelein RA, Berkhout B, Overbeek GP, van Duin J (1983). Effect of the sequences upstream from the ribosome-binding site on the yield of protein from the cloned gene for phage MS2 coat protein. Gene.

[2] Peabody DS (1990). Translational repression by bacteriophage MS2 coat protein expressed from a plasmid. A system for genetic analysis of a protein–RNA interaction. J Biol Chem.

[3] Kozlovskaya TM, Pumpen PP, Dreilina DE, Tsimanis AJ, Ose VP, Tsibinogin VV, Gren EJ (1986). Formation of capsid-like structures as a result of expression of coat protein gene of RNA phage fr. Dokl Akad Nauk SSSR.

[4] Kozlovska TM, Cielens I, Dreilinna D, Dislers A, Baumanis V, Ose V, Pumpens P (1993). Recombinant RNA phage Q beta capsid particles synthesized and self-assembled in *Escherichia coli*. Gene.

[5] Tissot AC, Maurer P, Nussberger J, Sabat R, Pfister T, Ignatenko S, Volk HD, Stocker H, Muller P, Jennings GT (2008). Effect of immunisation against angiotensin II with CYT006-AngQb on ambulatory blood pressure: a double-blind, randomised, placebo-controlled phase IIa study. Lancet.

[6] Beeh KM, Kanniess F, Wagner F, Schilder C, Naudts I, Hammann-Haenni A, Willers J, Stocker H, Mueller P, Bachmann MF, Renner WA (2013). The novel TLR-9 agonist QbG10 shows clinical efficacy in persistent allergic asthma. J Allergy Clin Immunol.

[7] Cornuz J, Zwahlen S, Jungi WF, Osterwalder J, Klingler K, van Melle G, Bangala Y, Guessous I, Muller P, Willers J (2008). A vaccine against nicotine for smoking cessation: a randomized controlled trial. PLoS ONE.

[8] Tumban E, Muttil P, Escobar CA, Peabody J, Wafula D, Peabody DS, Chackerian B (2015). Preclinical refinements of a broadly protective VLP-based HPV vaccine targeting the minor capsid protein, L2. Vaccine.

[9] Spohn G, Jennings GT, Martina BE, Keller I, Beck M, Pumpens P, Osterhaus AD, Bachmann MF (2010). A VLP-based vaccine targeting domain III of the West Nile virus E protein protects from lethal infection in mice. Virol J.

[10] Pumpens P, Renhofa R, Dishlers A, Kozlovska T, Ose V, Pushko P, Tars K, Grens E, Bachmann MF (2016). The True story and advantages of RNA phage capsids as nanotools. Intervirology.

[11] Rohovie MJ, Nagasawa M, Swartz JR (2017). Virus-like particles: next-generation nanoparticles for targeted therapeutic delivery. Bioeng Transl Med.

[12] Peabody DS, Manifold-Wheeler B, Medford A, Jordan SK (2008). do Carmo Caldeira J, Chackerian B: immunogenic display of diverse peptides on virus-like particles of RNA phage MS2. J Mol Biol.

[13] Bardwell VJ, Wickens M (1990). Purification of RNA and RNA-protein complexes by an R17 coat protein affinity method. Nucleic Acids Res.

[14] Tsai BP, Wang X, Huang L, Waterman ML (2011). Quantitative profiling of in vivo-assembled RNA-protein complexes using a novel integrated proteomic approach. Mol Cell Proteomics.

[15] Bertrand E, Chartrand P, Schaefer M, Shenoy SM, Singer RH, Long RM (1998). Localization of ASH1 mRNA particles in living yeast. Mol Cell.

[16] Klovins J, Overbeek GP, van den Worm SH, Ackermann HW, van Duin J (2002). Nucleotide sequence of a ssRNA phage from *Acinetobacter*: kinship to coliphages. J Gen Virol.

[17] Kazaks A, Voronkova T, Rumnieks J, Dishlers A, Tars K (2011). Genome structure of *Caulobacter* phage phiCb5. J Virol.

[18] Greninger AL, DeRisi JL (2015). Draft genome sequences of Leviviridae RNA phages EC and MB recovered from San Francisco wastewater. Genome Announc.

[19] Krishnamurthy SR, Janowski AB, Zhao G, Barouch D, Wang D (2016). Hyperexpansion of RNA bacteriophage diversity. PLoS Biol.

[20] Shi M, Lin XD, Tian JH, Chen LJ, Chen X, Li CX, Qin XC, Li J, Cao JP, Eden JS (2016). Redefining the invertebrate RNA virosphere. Nature.

[21] Rumnieks J, Tars K (2012). Diversity of pili-specific bacteriophages: genome sequence of IncM plasmid-dependent RNA phage M. BMC Microbiol.

[22] Kannoly S, Shao Y, Wang IN (2012). Rethinking the evolution of single-stranded RNA (ssRNA) bacteriophages based on genomic sequences and characterizations of two R-plasmid-dependent ssRNA phages, C-1 and Hgal1. J Bacteriol.

[23] Ruokoranta TM, Grahn AM, Ravantti JJ, Poranen MM, Bamford DH (2006). Complete genome sequence of the broad host range single-stranded RNA phage PRR1 places it in the *Levivirus* genus with characteristics shared with Alloleviviruses. J Virol.

[24] Min Jou W, Haegeman G, Ysebaert M, Fiers W (1972). Nucleotide sequence of the gene coding for the bacteriophage MS2 coat protein. Nature.

[25] Olsthoorn RCL, Garde G, Dayhuff T, Atkins JF, van Duin J (1995). Nucleotide sequences of a single-stranded RNA phage from *Pseudomonas aeruginosa*: kinship to coliphages and conservation of regulatory RNA structures. Virology.

[26] Persson M, Tars K, Liljas L (2008). The capsid of the small RNA phage PRR1 is stabilized by metal ions. J Mol Biol.

[27] Plevka P, Kazaks A, Voronkova T, Kotelovica S, Dishlers A, Liljas L, Tars K (2009). The structure of bacteriophage phiCb5 reveals a role of the RNA genome and metal ions in particle stability and assembly. J Mol Biol.

[28] Golmohammadi R, Fridborg K, Bundule M, Valegård K, Liljas L (1996). The crystal structure of bacteriophage Qb at 3.5 Å resolution. Structure.

[29] Tars K, Fridborg K, Bundule M, Liljas L (2000). Crystal structure of phage PP7 from *Pseudomonas aeruginosa* at 3.7 Å resolution. Virology.

[30] Shishovs M, Rumnieks J, Diebolder C, Jaudzems K, Andreas LB, Stanek J, Kazaks A, Kotelovica S, Akopjana I, Pintacuda G (2016). Structure of AP205 coat protein reveals circular permutation in ssRNA bacteriophages. J Mol Biol.

[31] Axblom C, Tars K, Fridborg K, Orna L, Bundule M, Liljas L (1998). Structure of phage fr capsids with a deletion in the FG loop: implications for viral assembly. Virology.

[32] Rumnieks J, Tars K (2018). Protein–RNA interactions in the single-stranded RNA bacteriophages. Subcell Biochem.

[33] Valegård K, Murray JB, Stockley PG, Stonehouse NJ, Liljas L (1994). Crystal structure of an RNA bacteriophage coat protein-operator complex. Nature.

[34] Persson M, Tars K, Liljas L (2013). PRR1 coat protein binding to its RNA translational operator. Acta Crystallogr D Biol Crystallogr.

[35] Rumnieks J, Tars K (2014). Crystal structure of the bacteriophage qbeta coat protein in complex with the RNA operator of the replicase gene. J Mol Biol.

[36] Chao JA, Patskovsky Y, Almo SC, Singer RH (2008). Structural basis for the coevolution of a viral RNA-protein complex. Nat Struct Mol Biol.

[37] Bendis I, Shapiro L (1970). Properties of *Caulobacter* ribonucleic acid bacteriophage phi Cb5. J Virol.

[38] Koning RI, Gomez-Blanco J, Akopjana I, Vargas J, Kazaks A, Tars K, Carazo JM, Koster AJ (2016). Asymmetric cryo-EM reconstruction of phage MS2 reveals genome structure in situ. Nat Commun.

[39] Dai X, Li Z, Lai M, Shu S, Du Y, Zhou ZH, Sun R (2017). In situ structures of the genome and genome-delivery apparatus in a single-stranded RNA virus. Nature.

[40] Gorzelnik KV, Cui Z, Reed CA, Jakana J, Young R, Zhang J (2016). Asymmetric cryo-EM structure of the canonical *Allolevivirus* Qbeta reveals a single maturation protein and the genomic ssRNA in situ. Proc Natl Acad Sci U S A.

[41] Rumnieks J, Tars K (2017). Crystal structure of the maturation protein from bacteriophage Qbeta. J Mol Biol.

[42] Twarock R, Bingham RJ, Dykeman EC, Stockley PG (2018). A modelling paradigm for RNA virus assembly. Curr Opin Virol.

[43] Camacho C, Coulouris G, Avagyan V, Ma N, Papadopoulos J, Bealer K, Madden TL (2009). BLAST+ : architecture and applications. BMC Bioinformatics.

[44] Felsenstein J. PHYLIP (Phylogeny Inference Package) version 3.6.: Department of Genome Sciences, University of Washington, Seattle.; 2005.

[45] Rambaut A. FigTree, version 1.4.3. http://tree.bio.ed.ac.uk/software/figtree. 2009.

