# Supplementary material for: Production and characterization of novel ssRNA bacteriophage virus-like particles from metagenomic sequencing data
Source: J Nanobiotechnology. 2019 May 13;17:61. doi: 10.1186/s12951-019-0497-8 (PMC6513524; doi:10.1186/s12951-019-0497-8)
Supplement: Supplementary file 2 — Additional file 2: Figure S1. SDS-PAGE analysis of the production level of the metagenomic ssRNA phage CPs. The samples represent total cellular protein content four hours after the CP expression was induced at 37 °C. 1 and M2—protein molecular weight markers (M1: bands of 10, 15, 25, 35, 40, 55, 70, 100, 130 and 180 kDa; M2: bands of 14, 18, 25, 35, 45, 66 and 116 kDa). Figure S2. Solubility of the metagenomic ssRNA phage CPs at 37°C and 15°C. The samples represent the soluble (s) and insoluble (d) cellular protein fractions after the CPs were produced at the indicated temperature. The proteins were produced at 15 °C only if they were completely insoluble at 37 °C. M2—lanes with MW marker—14, 18, 25, 35, 45, 66 and 116 kDa. M1 and M2—protein molecular weight markers (M1: bands of 10, 15, 25, 35, 40, 55, 70, 100, 130 and 180 kDa; M2: bands of 14, 18, 25, 35, 45, 66 and 116 kDa). Figure S3. Predicted RNA hairpin structures at the beginning of the replicase gene in the metagenomic ssRNA phage genome sequences. A region flanking 20 nucleotides in each direction from the first nucleotide of the replicase gene was used for the prediction. [file 12951_2019_497_MOESM2_ESM.pdf]

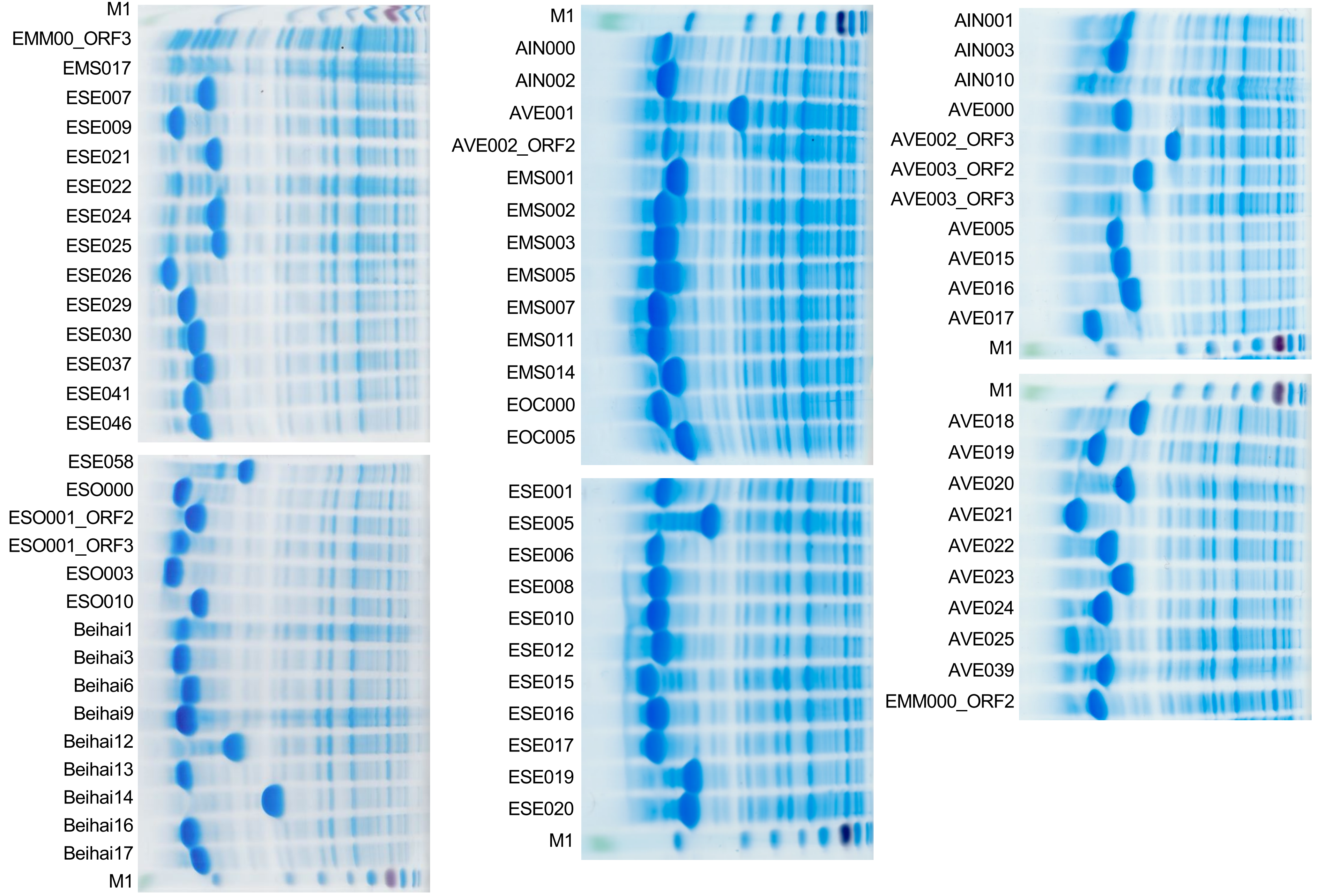

M1
EMM00_ORF3
EMS017
ESE007
ESE009
ESE021
ESE022
ESE024
ESE025
ESE026
ESE029
ESE030
ESE037
ESE041
ESE046
ESE058
ESO000
ESO001_ORF2
ESO001_ORF3
ESO003
ESO010
Beihai1
Beihai3
Beihai6
Beihai9
Beihai12
Beihai13
Beihai14
Beihai16
Beihai17
M1
M1
AIN000
AIN002
AVE001
AVE002_ORF2
EMS001
EMS002
EMS003
EMS005
EMS007
EMS011
EMS014
EOC000
EOC005
ESE001
ESE005
ESE006
ESE008
ESE010
ESE012
ESE015
ESE016
ESE017
ESE019
ESE020
M1
AIN001
AIN003
AIN010
AVE000
AVE002_ORF3
AVE003_ORF2
AVE003_ORF3
AVE005
AVE015
AVE016
AVE017
M1
M1
AVE018
AVE019
AVE020
AVE021
AVE022
AVE023
AVE024
AVE025
AVE039
EMM000_ORF2

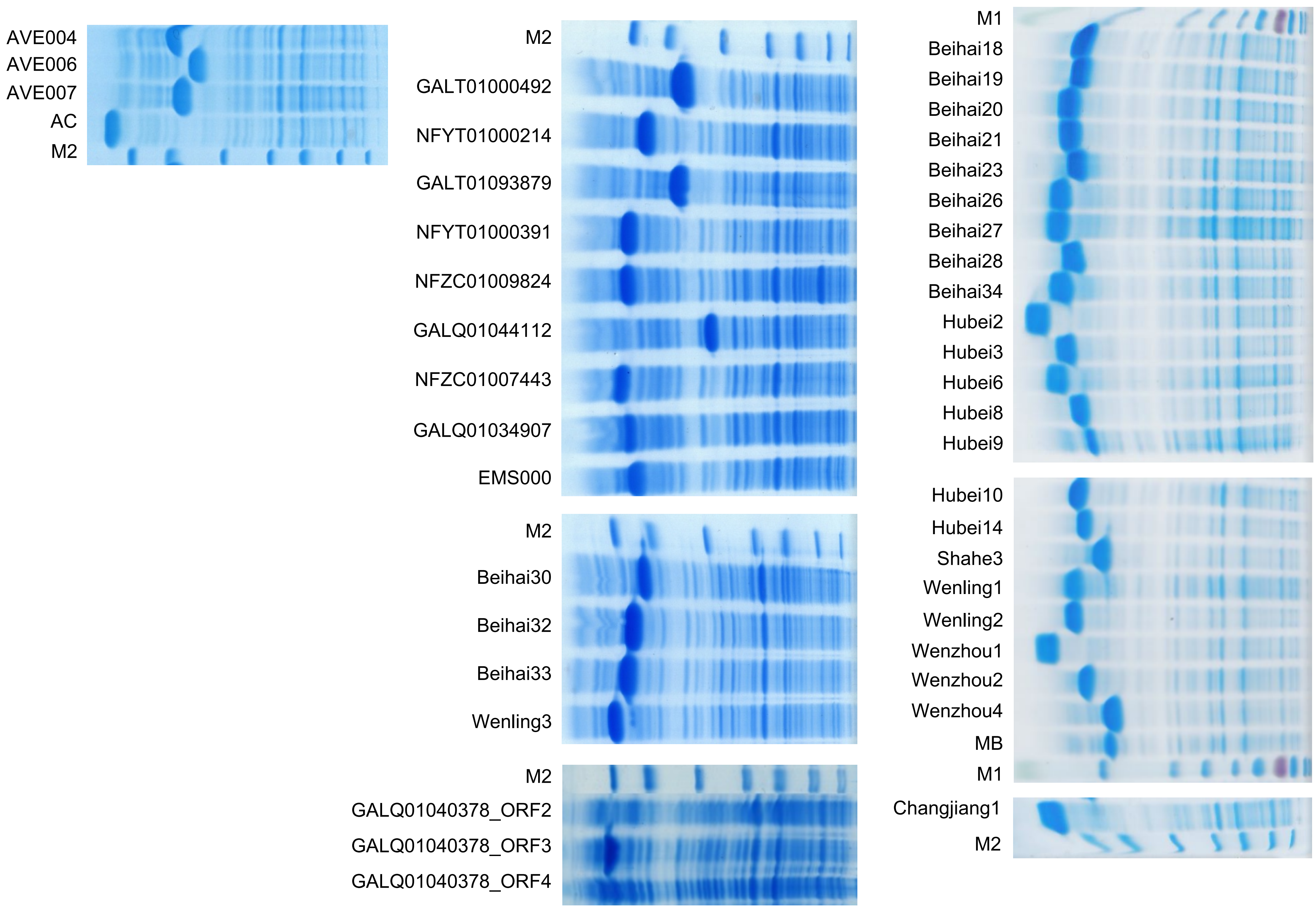
M1
Beihai18
Beihai19
Beihai20
Beihai21
Beihai23
Beihai26
Beihai27
Beihai28
Beihai34
Hubei2
Hubei3
Hubei6
Hubei8
Hubei9
Hubei10
Hubei14
Shahe3
Wenling1
Wenling2
Wenzhou1
Wenzhou2
Wenzhou4
MB
M1
Changjiang1
M2
M2
GALT01000492
NFYT01000214
GALT01093879
NFYT01000391
NFZC01009824
GALQ01044112
NFZC01007443
GALQ01034907
EMS000
M2
Beihai30
Beihai32
Beihai33
Wenling3
M2
GALQ01040378_ORF2
GALQ01040378_ORF3
GALQ01040378_ORF4
AVE004
AVE006
AVE007
AC
M2

Figure S1. SDS-PAGE analysis of the production level of the metagenomic ssRNA phage CPs. The samples represent total cellular protein content four hours after the CP expression was induced at 37 °C. 1 and M2 – protein molecular weight markers (M1: bands of 10, 15, 25, 35, 40, 55, 70, 100, 130 and 180 kDa; M2: bands of 14, 18, 25, 35, 45, 66 and 116 kDa).

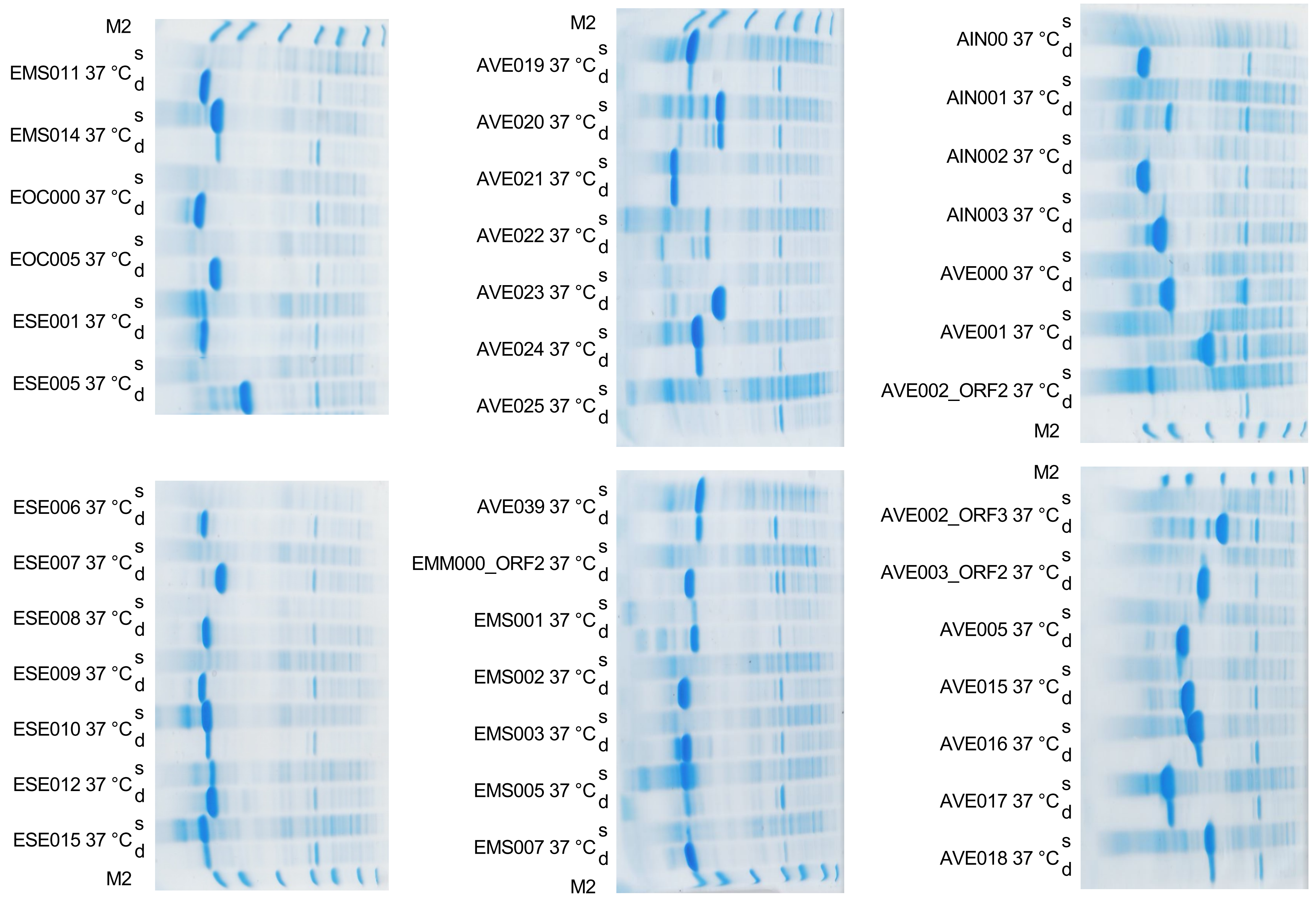
M2
EMS011 37 °C s d
EMS014 37 °C s d
EOC000 37 °C s d
EOC005 37 °C s d
ESE001 37 °C s d
ESE005 37 °C s d
M2
AVE019 37 °C s d
AVE020 37 °C s d
AVE021 37 °C s d
AVE022 37 °C s d
AVE023 37 °C s d
AVE024 37 °C s d
AVE025 37 °C s d
AIN00 37 °C s d
AIN001 37 °C s d
AIN002 37 °C s d
AIN003 37 °C s d
AVE000 37 °C s d
AVE001 37 °C s d
AVE002_ORF2 37 °C s d
M2
ESE006 37 °C s d
ESE007 37 °C s d
ESE008 37 °C s d
ESE009 37 °C s d
ESE010 37 °C s d
ESE012 37 °C s d
ESE015 37 °C s d
M2
AVE039 37 °C s d
EMM000_ORF2 37 °C s d
EMS001 37 °C s d
EMS002 37 °C s d
EMS003 37 °C s d
EMS005 37 °C s d
EMS007 37 °C s d
M2
M2
AVE002_ORF3 37 °C s d
AVE003_ORF2 37 °C s d
AVE005 37 °C s d
AVE015 37 °C s d
AVE016 37 °C s d
AVE017 37 °C s d
AVE018 37 °C s d

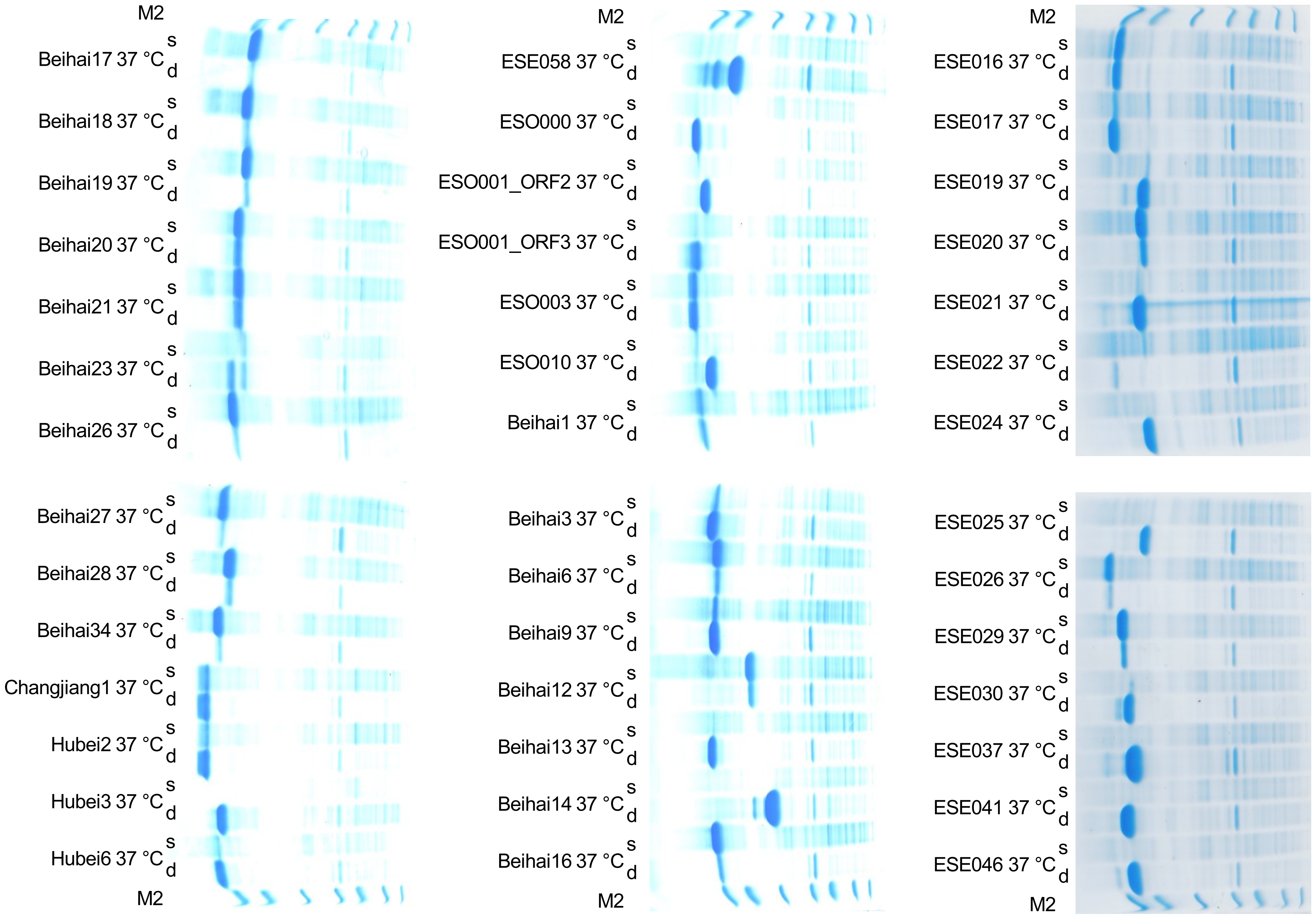

M2
Beihai17 37 °C s d
Beihai18 37 °C s d
Beihai19 37 °C s d
Beihai20 37 °C s d
Beihai21 37 °C s d
Beihai23 37 °C s d
Beihai26 37 °C s d
Beihai27 37 °C s d
Beihai28 37 °C s d
Beihai34 37 °C s d
Changjiang1 37 °C s d
Hubei2 37 °C s d
Hubei3 37 °C s d
Hubei6 37 °C s d
M2
M2
ESE058 37 °C s d
ESO000 37 °C s d
ESO001_ORF2 37 °C s d
ESO001_ORF3 37 °C s d
ESO003 37 °C s d
ESO010 37 °C s d
Beihai1 37 °C s d
Beihai3 37 °C s d
Beihai6 37 °C s d
Beihai9 37 °C s d
Beihai12 37 °C s d
Beihai13 37 °C s d
Beihai14 37 °C s d
Beihai16 37 °C s d
M2
M2
ESE016 37 °C s d
ESE017 37 °C s d
ESE019 37 °C s d
ESE020 37 °C s d
ESE021 37 °C s d
ESE022 37 °C s d
ESE024 37 °C s d
ESE025 37 °C s d
ESE026 37 °C s d
ESE029 37 °C s d
ESE030 37 °C s d
ESE037 37 °C s d
ESE041 37 °C s d
ESE046 37 °C s d
M2

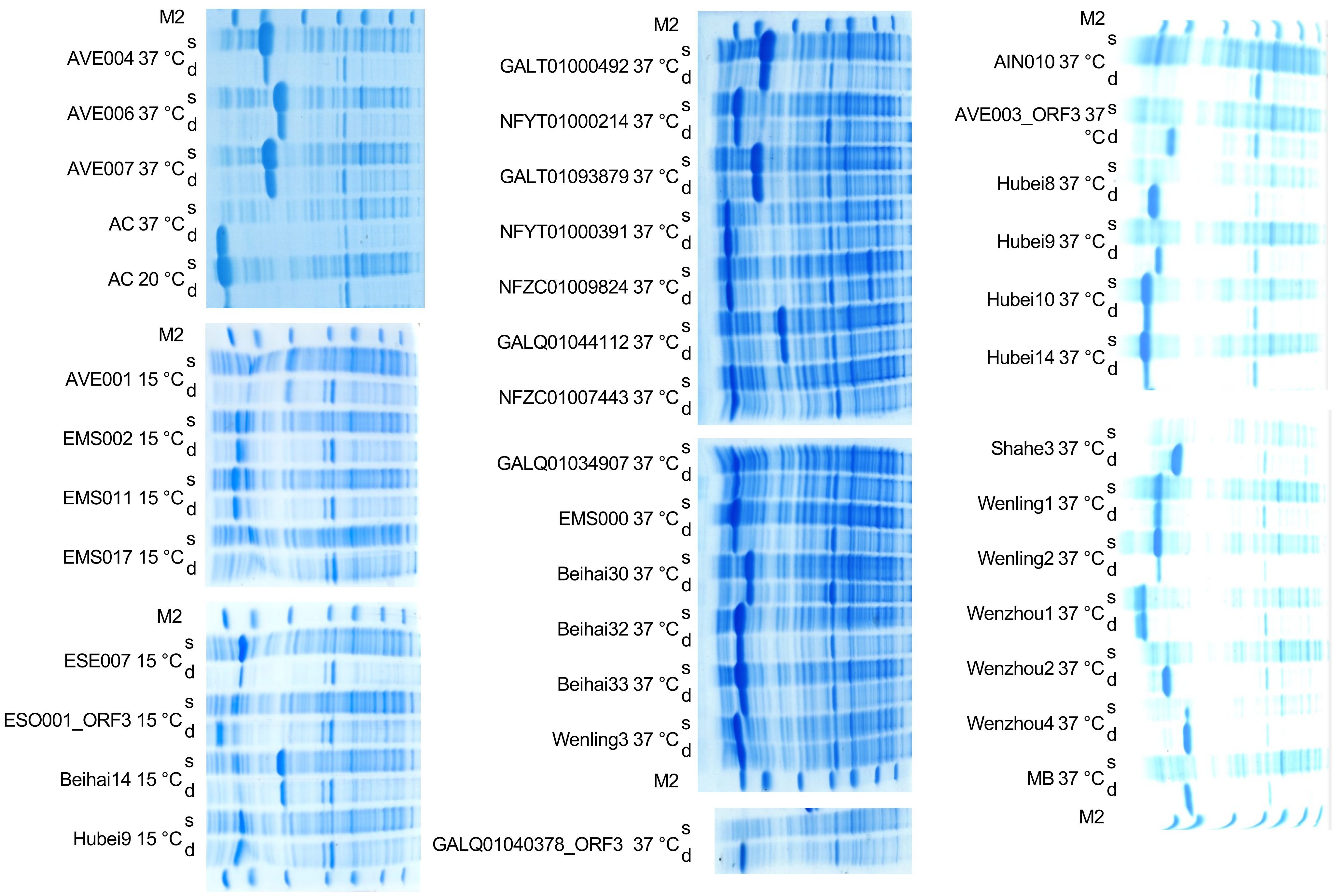
M2
AVE004 37 °C s d
AVE006 37 °C s d
AVE007 37 °C s d
AC 37 °C s d
AC 20 °C s d
M2
AVE001 15 °C s d
EMS002 15 °C s d
EMS011 15 °C s d
EMS017 15 °C s d
M2
ESE007 15 °C s d
ESO001_ORF3 15 °C s d
Beihai14 15 °C s d
Hubei9 15 °C s d
M2
GALT01000492 37 °C s d
NFYT01000214 37 °C s d
GALT01093879 37 °C s d
NFYT01000391 37 °C s d
NFZC01009824 37 °C s d
GALQ01044112 37 °C s d
NFZC01007443 37 °C s d
GALQ01034907 37 °C s d
EMS000 37 °C s d
Beihai30 37 °C s d
Beihai32 37 °C s d
Beihai33 37 °C s d
Wenling3 37 °C s d
M2
GALQ01040378_ORF3 37 °C s d
M2
AIN010 37 °C s d
AVE003_ORF3 37 °C s d
Hubei8 37 °C s d
Hubei9 37 °C s d
Hubei10 37 °C s d
Hubei14 37 °C s d
Shahe3 37 °C s d
Wenling1 37 °C s d
Wenling2 37 °C s d
Wenzhou1 37 °C s d
Wenzhou2 37 °C s d
Wenzhou4 37 °C s d
MB 37 °C s d
M2

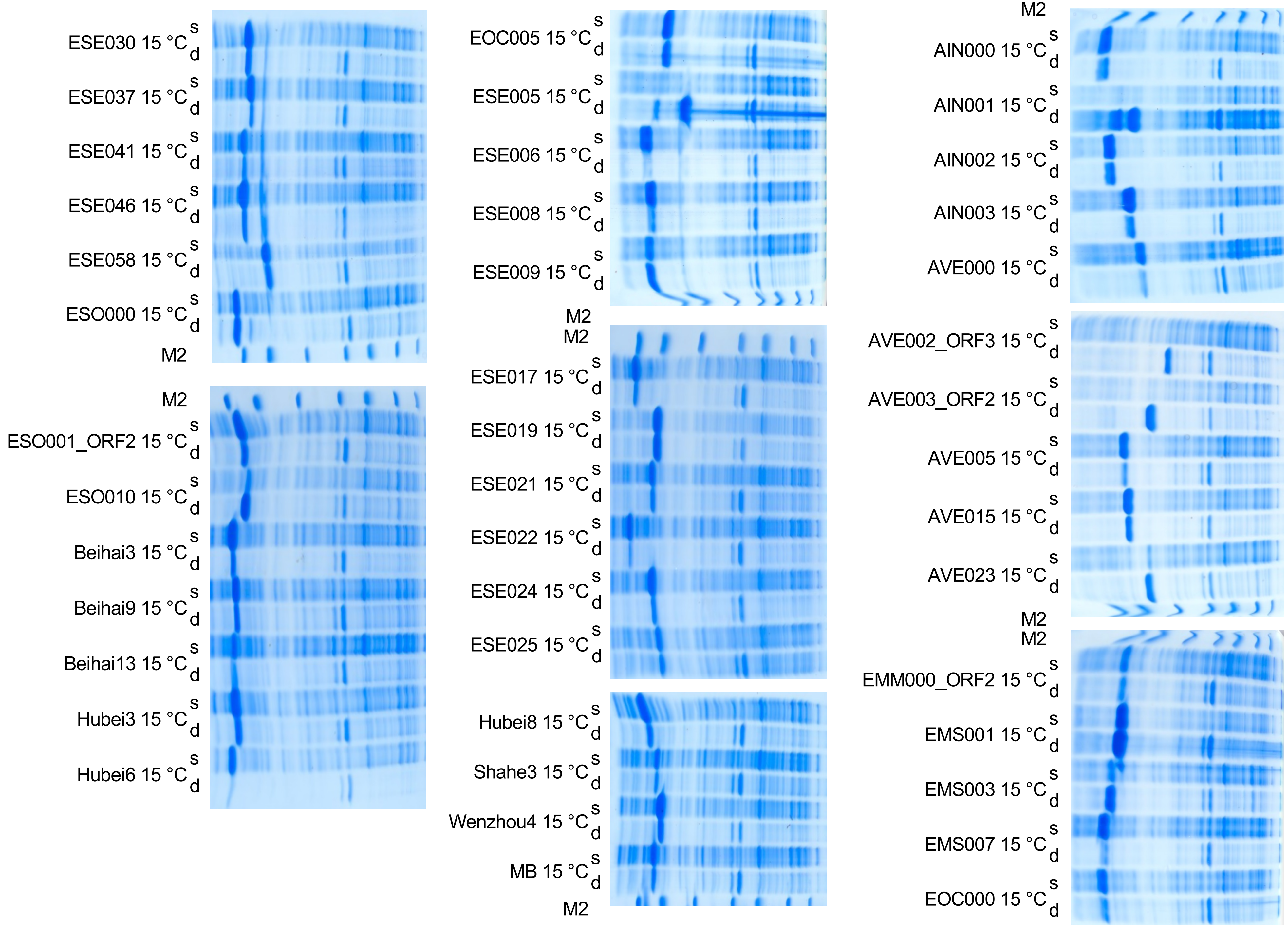
M2
AIN000 15 °C s d
AIN001 15 °C s d
AIN002 15 °C s d
AIN003 15 °C s d
AVE000 15 °C s d
AVE002_ORF3 15 °C s d
AVE003_ORF2 15 °C s d
AVE005 15 °C s d
AVE015 15 °C s d
AVE023 15 °C s d
M2
M2
EMM000_ORF2 15 °C s d
EMS001 15 °C s d
EMS003 15 °C s d
EMS007 15 °C s d
EOC000 15 °C s d
EOC005 15 °C s d
ESE005 15 °C s d
ESE006 15 °C s d
ESE008 15 °C s d
ESE009 15 °C s d
M2
M2
ESE017 15 °C s d
ESE019 15 °C s d
ESE021 15 °C s d
ESE022 15 °C s d
ESE024 15 °C s d
ESE025 15 °C s d
Hubei8 15 °C s d
Shahe3 15 °C s d
Wenzhou4 15 °C s d
MB 15 °C s d
M2
ESE030 15 °C s d
ESE037 15 °C s d
ESE041 15 °C s d
ESE046 15 °C s d
ESE058 15 °C s d
ESO000 15 °C s d
M2
M2
ESO001_ORF2 15 °C s d
ESO010 15 °C s d
Beihai3 15 °C s d
Beihai9 15 °C s d
Beihai13 15 °C s d
Hubei3 15 °C s d
Hubei6 15 °C s d

Figure S2. Solubility of the metagenomic ssRNA phage CPs at 37°C and 15°C. The samples represent the soluble (s) and insoluble (d) cellular protein fractions after the CPs were produced at the indicated temperature. The proteins were produced at 15 °C only if they were completely insoluble at 37 °C. M2 – lanes with MW marker - 14, 18, 25, 35, 45, 66 and 116 kDa. M1 and M2 – protein molecular weight markers (M1: bands of 10, 15, 25, 35, 40, 55, 70, 100, 130 and 180 kDa; M2: bands of 14, 18, 25, 35, 45, 66 and 116 kDa).

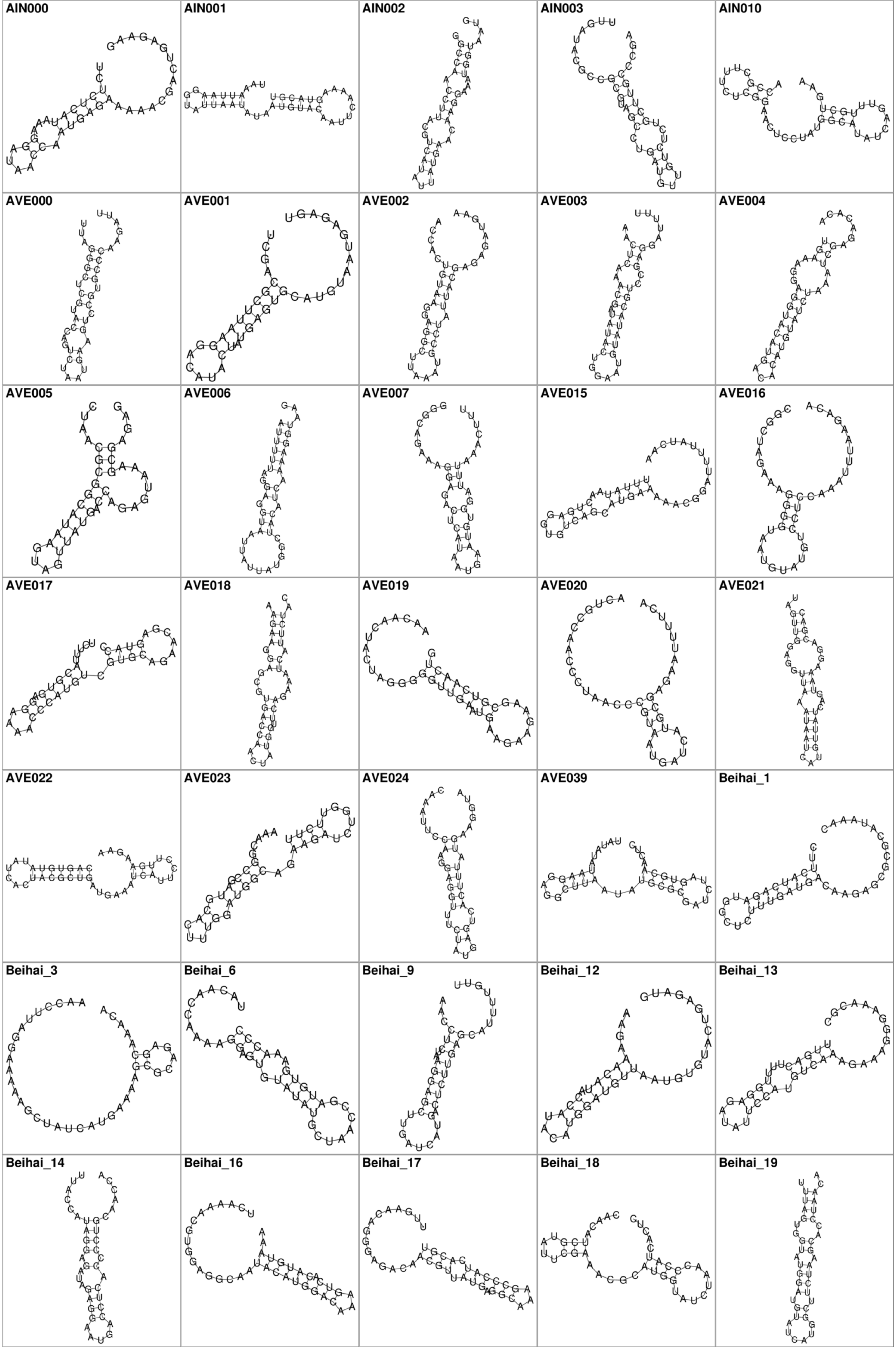
AIN000
AIN001
AIN002
AIN003
AIN010
AVE000
AVE001
AVE002
AVE003
AVE004
AVE005
AVE006
AVE007
AVE015
AVE016
AVE017
AVE018
AVE019
AVE020
AVE021
AVE022
AVE023
AVE024
AVE039
Beihai_1
Beihai_3
Beihai_6
Beihai_9
Beihai_12
Beihai_13
Beihai_14
Beihai_16
Beihai_17
Beihai_18
Beihai_19

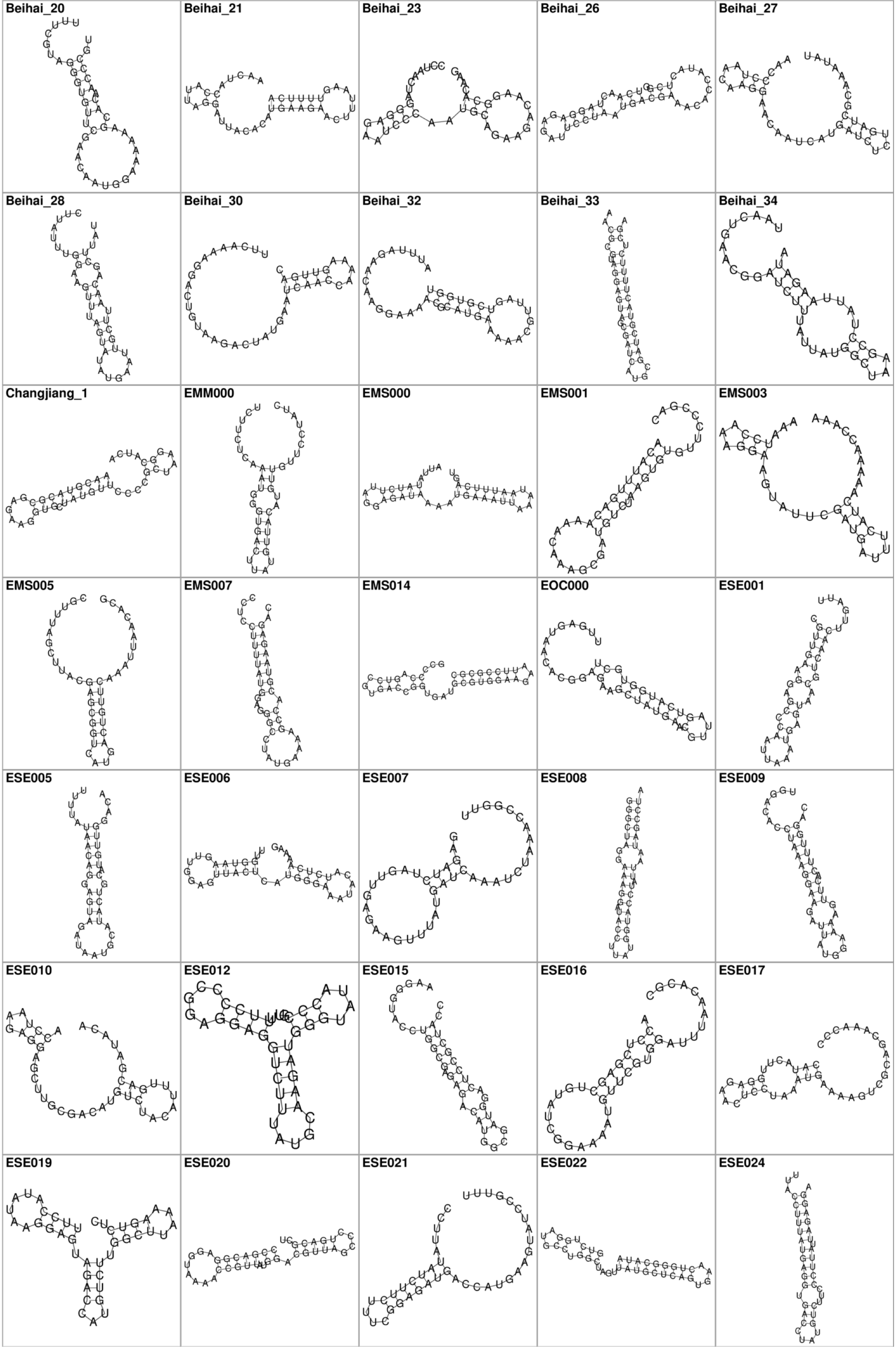
Beihai_20
Beihai_21
Beihai_23
Beihai_26
Beihai_27
Beihai_28
Beihai_30
Beihai_32
Beihai_33
Beihai_34
Changjiang_1
EMM000
EMS000
EMS001
EMS003
EMS005
EMS007
EMS014
EOC000
ESE001
ESE005
ESE006
ESE007
ESE008
ESE009
ESE010
ESE012
ESE015
ESE016
ESE017
ESE019
ESE020
ESE021
ESE022
ESE024

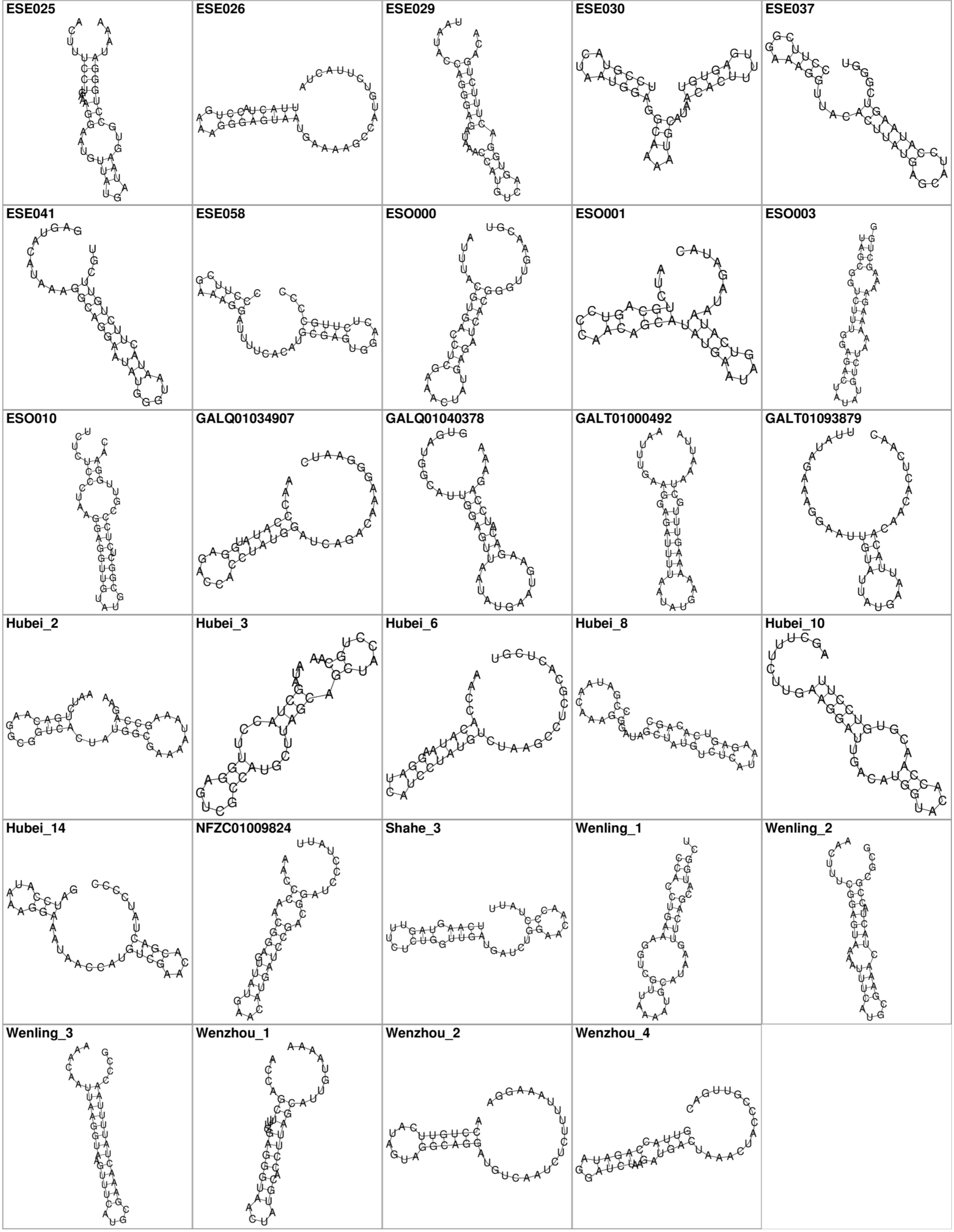

Figure S3. Predicted RNA hairpin structures at the beginning of the replicase gene in the metagenomic ssRNA phage genome sequences. A region flanking 20 nucleotides in each direction from the first nucleotide of the replicase gene was used for the prediction.
